# Modulation of Enhancer Looping and Differential Gene Targeting by Epstein-Barr Virus Transcription Factors Directs Cellular Reprogramming

**DOI:** 10.1371/journal.ppat.1003636

**Published:** 2013-09-12

**Authors:** Michael J. McClellan, C. David Wood, Opeoluwa Ojeniyi, Tim J. Cooper, Aditi Kanhere, Aaron Arvey, Helen M. Webb, Richard D. Palermo, Marie L. Harth-Hertle, Bettina Kempkes, Richard G. Jenner, Michelle J. West

**Affiliations:** 1 School of Life Sciences, John Maynard-Smith Building, University of Sussex, Falmer, Brighton, United Kingdom; 2 MRC Centre for Medical Molecular Virology, Division of Infection and Immunity, Paul O'Gorman Building, University College London, London, United Kingdom; 3 Memorial Sloan-Kettering Cancer Center, New York, New York, United States of America; 4 Department of Gene Vectors, Helmholtz Center Munich, German Research Center for Environmental Health, Munich, Germany; Wistar Institute, United States of America

## Abstract

Epstein-Barr virus (EBV) epigenetically reprogrammes B-lymphocytes to drive immortalization and facilitate viral persistence. Host-cell transcription is perturbed principally through the actions of EBV EBNA 2, 3A, 3B and 3C, with cellular genes deregulated by specific combinations of these EBNAs through unknown mechanisms. Comparing human genome binding by these viral transcription factors, we discovered that 25% of binding sites were shared by EBNA 2 and the EBNA 3s and were located predominantly in enhancers. Moreover, 80% of potential EBNA 3A, 3B or 3C target genes were also targeted by EBNA 2, implicating extensive interplay between EBNA 2 and 3 proteins in cellular reprogramming. Investigating shared enhancer sites neighbouring two new targets (*WEE1* and *CTBP2*) we discovered that EBNA 3 proteins repress transcription by modulating enhancer-promoter loop formation to establish repressive chromatin hubs or prevent assembly of active hubs. Re-ChIP analysis revealed that EBNA 2 and 3 proteins do not bind simultaneously at shared sites but compete for binding thereby modulating enhancer-promoter interactions. At an EBNA 3-only intergenic enhancer site between *ADAM28* and *ADAMDEC1* EBNA 3C was also able to independently direct epigenetic repression of both genes through enhancer-promoter looping. Significantly, studying shared or unique EBNA 3 binding sites at *WEE1*, *CTBP2, ITGAL* (LFA-1 alpha chain), *BCL2L11* (Bim) and the *ADAMs*, we also discovered that different sets of EBNA 3 proteins bind regulatory elements in a gene and cell-type specific manner. Binding profiles correlated with the effects of individual EBNA 3 proteins on the expression of these genes, providing a molecular basis for the targeting of different sets of cellular genes by the EBNA 3s. Our results therefore highlight the influence of the genomic and cellular context in determining the specificity of gene deregulation by EBV and provide a paradigm for host-cell reprogramming through modulation of enhancer-promoter interactions by viral transcription factors.

## Author Summary

Epstein-Barr virus (EBV) is associated with numerous cancers. The ability of the virus to infect B-cells and convert them from short-lived into immortal cells is the key to its cancer-promoting properties. A small number of EBV transcription factors are required for immortalization and act in concert to drive cell growth by deregulating the expression of cellular genes through largely unknown mechanisms. We have demonstrated that four of these key transcription factors function cooperatively by targeting common genes via long-range enhancer elements and modulating their looping interactions with gene promoters. Specifically we show that gene repression by the EBV EBNA 3 family of proteins can be mediated through the modulation of enhancer-promoter looping. Our results also reveal that different subsets of EBNA 3 proteins are bound at different genes and that this differential binding can vary in lymphoma cells compared to cells immortalized in culture, indicating that cell-background-specific gene regulation may be important in lymphoma development. Our results demonstrate how cellular genes can be deregulated by an oncogenic virus through modulation of enhancer-promoter looping with the specificity of binding by viral transcription factors controlling cellular reprogramming in a gene and cell-type specific manner.

## Introduction

Epstein-Barr virus (EBV) was discovered in cells cultured from a Burkitt's lymphoma (BL) biopsy in 1964 [1] and has since been associated with the development of numerous cancers including Hodgkin's disease, post-transplant lymphoma, certain natural killer and T-cell lymphomas and the epithelial cell tumour nasopharyngeal carcinoma. EBV immortalizes host B lymphocytes generating latently infected lymphoblastoid cell lines (LCLs) that proliferate indefinitely in culture. LCLs express 9 viral latent proteins: six Epstein-Barr nuclear antigens (EBNAs) and three latent membrane proteins (LMPs). All EBNAs possess transcriptional regulatory functions and EBNA 2 and the EBNA 3 family of proteins (3A, 3B and 3C) function as key regulators of viral and cellular transcription in immortalized cells.

EBNA 2 is essential for initial B-cell immortalization by EBV and for the continuous growth of EBV transformed cell lines [2]–[3]. EBNA 2 functions as the master controller of latent viral gene transcription and activates the EBV C promoter that drives production of the long pre-mRNA encoding all EBNAs, in addition to the promoters of the LMP genes [4]–[7]. EBNA 2 also activates transcription of numerous cellular genes involved in growth control and B-cell activation including *MYC*, *RUNX3, CD23, CD21* and *FGR* [8]–[11]. EBNA 2 cannot bind DNA directly and interacts with target genes via cellular transcription factors that include RBP-Jκ (C promoter binding factor 1, CBF1) and PU.1 (Spi-1) [12]–[15]. ATF-2/c-Jun heterodimers and EBF1 have also been implicated in EBNA 2 activation of the EBV LMP1 promoter [16]–[17] and AUF1 was identified as the CBF2 factor required for efficient activation of the EBV C promoter [18]. EBNA 2 mediates transcriptional activation through an acidic activation domain [19]–[20] and interacts with a number of general transcription factors including TFIIB, TAF40, the TFIIH p62 and p80 subunits and a TFIIE–associated protein [21]–[23]. Transcriptional activation by EBNA 2 is also mediated through association with histone acetyltransferases [24] and chromatin remodelling complexes [25]–[26]. An additional EBNA, EBNA-leader protein (EBNA-LP) plays a role in augmenting transcriptional activation by EBNA 2. Expression of EBNA-LP enhances EBNA 2-induced activation of the viral LMP1 promoter via RBP-Jκ sites and EBNA 2 and EBNA-LP cooperate to induce cyclin D2 expression in resting B-cells [27]–[29]. EBNA-LP coactivation may be mediated through relocalization of histone deacetylases from EBNA 2 activated promoters [30].

The genes encoding the EBNA 3 family of transcriptional regulators (3A, 3B and 3C) probably arose as a result of gene duplication, since they are tandemly arranged in the EBV genome, consist of a short 5′ and long 3′ exon and display an albeit low degree of amino-acid identity. EBNA 3A and 3C were shown to be required for immortalization of B-cells by EBV [31], although more recently short-lived EBV-infected cell lines have been generated from EBNA 3A knock-out viruses [32]. These data, combined with studies using cell lines infected with viruses expressing conditionally active forms of EBNA 3A or 3C, indicate that the continued proliferation of immortalized cells is dependent on 3A and 3C function [33]–[34]. EBNA 3B however, is not essential for *in vitro* immortalization [35] but confers a tumour suppressive function *in vivo* [36].

Gene expression analysis demonstrates that EBNA 3A, 3B and 3C function as both activators and repressors of cellular gene expression and transcriptional repression and activation domains of EBNA 3C have been identified using Gal4-fusion protein assays [32], [37]–[41]. A well-documented repressive function of the EBNA 3 proteins involves the inhibition of EBNA 2 activation through association with the EBNA 2 targeting partner RBP-Jκ. All EBNA 3 proteins can bind RBP-Jκ and inhibit EBNA 2 activation via RBP-Jκ sites in reporter assays, although loss of EBNA 3A and 3C function does not increase EBNA 2 activation of some key target promoters containing RBP-Jκ sites in infected cells [33]–[34], [42]–[44]. In addition to RBP-Jκ, EBNA 3C also binds to the PU.1 transcription factor implicated in EBNA 2 activation of the viral LMP1 promoter [45]. In fact EBNA 3C is able to coactivate the LMP1 promoter with EBNA 2 in a manner dependent on the PU.1 binding motif [45]. Transcriptional repression by EBNA 3A and 3C can also be mediated through interactions with, or recruitment of, multiple transcriptional co-repressors including C-terminal binding protein (CtBP), histone deacetylases and polycomb repressor complexes [46]–[50]. EBNA 3C also associates with the histone acetyltransferase p300, implicating histone acetylation in EBNA 3C-mediated transcriptional activation [51].

Interestingly, gene expression profiling reveals extensive overlap in the cellular genes regulated by EBNA 3 proteins [37]–[38]. Roughly half of the genes affected by the loss of an individual EBNA 3 protein in studies using EBV-negative BL cell lines infected with recombinant knock-out EBVs were also found to be deregulated in the absence of another EBNA 3 family member [38]. Co-operative gene regulation by the EBNA 3s is exemplified in the regulation of genes encoding two key regulators of immortalized cell growth/survival, the pro-apoptotic protein Bim (*BCL2L11*) and the cyclin-dependent kinase inhibitor p16^INK4A^ (*CDKN2A*) [52]–[55]. These genes are repressed through the concerted actions of EBNA 3A and 3C via the polycomb-associated trimethylation of lysine 27 on histone H3 [48], [53], [55]. Overlap in cellular gene regulation between EBNA 3 proteins and EBNA 2 is also evident. For example, a recent study found that 25% of EBNA 3A-regulated genes identified in LCLs generated from EBNA 3A mutant viruses were also regulated by EBNA 2, in either a co-operative (9%) or antagonistic manner (16%) [32]. Moreover, when we compared the EBNA 3C-regulated genes identified in our own microarray analysis [37] with genes identified in five EBNA 2 gene expression arrays [11], [56]–[59], we found that 27% of genes were also regulated by EBNA 2. Despite the identification of significant numbers of cellular genes regulated by EBNA 2, 3A, 3B and 3C, the mechanism through which the majority of these genes are targeted and epigenetically reprogrammed remains unclear. Interestingly, our own ChIP-sequencing analysis of human genome binding by the EBNA 3s and analysis of EBNA 2 binding by Zhao and colleagues has highlighted a role for long-range enhancer elements in cellular gene deregulation by these EBV transcription factors [17], [37].

We set out to investigate the mechanism of cooperative cellular gene deregulation by EBNA 2, 3A, 3B and 3C and the role of long-range enhancers in host-cell reprogramming by EBV. Significantly, studying regulation via long-range regulatory elements we found that EBNA 3 proteins repress cellular gene transcription by modulating the formation of chromatin loops between enhancers and promoters. Importantly, our studies also determined that gene control by different members of the EBNA 3 transcription factor family is mediated by gene and cell-type specific binding of subsets of these factors. Our studies therefore indicate that transcriptional reprogramming by EBV can be gene and host-cell-specific and involves exploitation and modulation of the three-dimensional chromatin architecture connecting long-range enhancers with gene promoters.

## Results

### EBNA 2 and 3 proteins target common sites and genes

**Figure 1:**
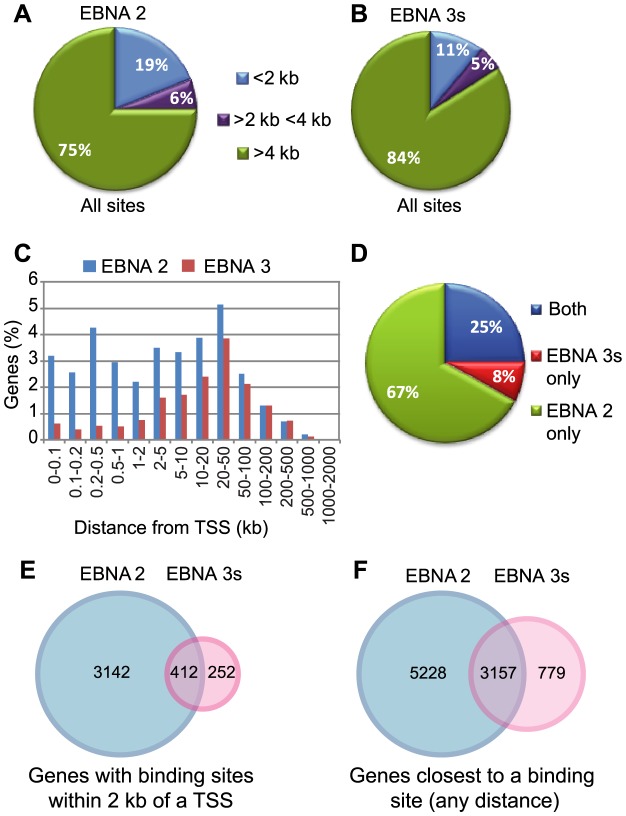
Analysis of ChIP-seq data for EBNA 2 and EBNA 3 proteins. (A) Pie chart showing the distribution of all significant binding sites for EBNA 2 relative to gene TSSs. (B) Distribution of EBNA 3 family binding sites. (C) The frequency of EBNA 2 or EBNA 3 protein binding sites plotted as distance from the TSS of the closest gene. (D) Pie chart showing the proportion of sites identified for EBNA 2 and EBNA 3 family proteins that are shared or unique. (E) Comparison of genes closest to EBNA 2 binding sites with genes closest to EBNA 3 binding sites for sites within 2 kb of a TSS. (F) Comparison of genes closest to an EBNA 2 or EBNA 3 binding site located any distance from a gene TSS.

To examine the mechanism of interplay in cellular gene reprogramming by EBNA 2, 3A, 3B and 3C proteins, we performed comparative analysis of the human genome regulatory elements targeted by these factors in a BL cell line expressing all EBV latent proteins (Mutu III) ([60] and Figure S1). Since EBNA 2 and 3 proteins do not possess direct DNA-binding activity and interact with their target sites through interactions with cellular DNA-binding proteins, the binding sites analysed in this study represent sites at which the EBNAs indirectly interact with DNA. ChIP-seq was carried out using an EBNA 2-specific monoclonal antibody and compared to ChIP-seq data generated in our laboratory using an anti-EBNA 3C polyclonal antibody that we found also independently precipitates EBNA 3A and 3B, albeit at a lower efficiency (Figure S2) [37] (ChIP-seq data are available through GEO Series accession number GSE47629). Our analysis identified 21,605 significant EBNA 2 binding sites and 7044 significant EBNA 3 binding sites in the human genome indicating that overall EBNA 3A, 3B and 3C proteins target fewer regulatory regions than EBNA 2. Our previous analysis of the human genome binding profile of the EBNA 3 proteins in the Mutu III cell line and recent analysis of EBNA 2 binding in an LCL carrying an EBNA 3B-deleted virus by Zhao *et al* revealed that only a small proportion of binding sites for these factors are proximal to gene transcription start sites (TSS) [17], [37]. Consistent with these observations, our analysis revealed that 75% of EBNA 2 sites and 84% of EBNA 3 sites were located distal (>4 kb) to TSSs (Figure 1A and B). Examination of the distances between genes and the closest binding sites for EBNA 2 and EBNA 3 proteins revealed that the closest EBNA 3 binding site was most often 10–50 kb from TSSs. In contrast, the closest EBNA 2 binding sites were found both proximal and distal to gene TSSs with similar frequency (Figure 1C). In conclusion, EBNA 2 and 3 proteins generally target distal regulatory elements rather than promoter sequences, with this being most apparent for the EBNA 3s.

We next considered how EBNA 2 and 3 binding patterns might be related. Comparing binding we detected considerable overlap in the regulatory elements targeted by these proteins, with 25% of all highly significant sites identified bound by both EBNA 2 and the EBNA 3s (Figure 1D). Surprisingly, EBNA 3-only sites constituted only 8% of the sites identified in this analysis (Figure 1D). These data point to a key role of EBNA 3 proteins in the coregulation of cellular gene expression with EBNA 2.

We next sought to identify the genes targeted by EBNA 2 and 3 proteins via the binding sites we had mapped. Looking at binding sites located within 2 kb of a gene TSS, we found that EBNA 2 was associated with 3554 genes and EBNA 3 with 664 genes, consistent with the smaller number of EBNA 3 binding sites in the genome. Comparing genes with EBNA 3 binding sites within 2 kb of the TSS with genes within 2 kb of EBNA 2 binding sites revealed that 62% (412/664) of EBNA 3 proximal target genes were also bound by EBNA 2 (Figure 1E). In fact for 411 of these 412 genes, the proximal EBNA 2 and 3 binding sites were overlapping. Using more relaxed criteria to associate a binding site with a gene, we also identified the genes that were closest to a binding site irrespective of the distance from the site. Using this approach, we found that 80% (3157/3937) of genes closest to an EBNA 3 binding site were also the closest genes to an EBNA 2 binding site. Taken together our analysis indicates that EBNA 2 and 3 proteins generally target the same cellular genes and that a major role of the EBNA 3 proteins is in the co-regulation of genes with EBNA 2.

### Comparison with gene expression array data links gene targeting with regulation

To obtain information on whether the potential gene targets we had identified through binding site analysis were regulated by EBNA 2 or EBNA 3 proteins, we examined data available from our own and other published gene expression array studies [11], [32], [37]–[39], [56]–[59], [61]–[62]. We found that 46% (299/654) of EBNA 2-regulated genes identified in these studies had EBNA 2 binding sites within 2 kb of a TSS. In contrast only 8% (199/2601) of documented EBNA 3-regulated genes had promoter-proximal EBNA 3 protein binding sites, likely reflecting the fact that gene regulation by the EBNA 3s is predominantly mediated via distal elements. Consistent with distal regulation of gene expression by the EBNA 3 proteins, the proportion of previously identified EBNA 3-regulated genes associated with an EBNA 3 binding site increased to 31% (802/2601) when we considered genes that were closest to a binding site, irrespective of the distance [32], [37]–[39], [61]–[62]. Concordantly, the proportion of previously identified EBNA 2-regulated target genes associated with an EBNA 2 binding site increased to 60% (393/654) when genes any distance from a binding site were included in the analysis.

### EBNA 2 and EBNA 3 binding sites colocalize with histone modifications found at regulatory regions

**Figure 2:**
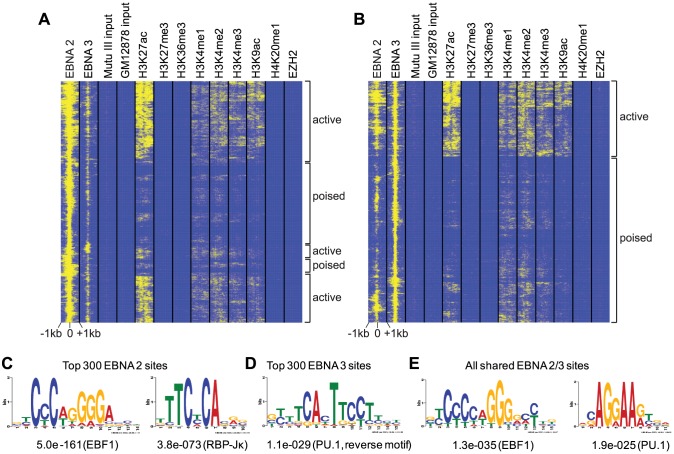
Colocalization of histone modifications and transcription factor binding at EBNA 2 and 3 binding sites. (A) Heatmap of EBNA 2, EBNA 3 and histone modification ChIP-seq signals at the top 1000 EBNA 2 binding sites. EBNA 2 and 3 ChIP-seq data from Mutu III BL cells was aggregated with ENCODE histone modification ChIP-seq data from the GM12878 LCL using hierarchical clustering. Each window displays the ChIP-seq signal −/+ 1 kb around the EBNA 2 binding site midpoint. Clusters of active enhancers (H3K4me1+, H3K27ac+) and poised enhancers (H3K4me1+, H3K27ac−) are indicated. (B) Heatmap of EBNA 3, EBNA 2 and histone modification ChIP-seq signals at the top 1000 EBNA 3 binding sites. (C) Position weight matrix and TF consensus prediction generated from unbiased motif searching using the top 300 EBNA 2 binding sites. Numbers show the p-value for site enrichment. (D) Position weight matrix derived from motif searching using the top 300 EBNA 3 family sites. (E) Position weight matrix for motif searching using all shared sites.

To obtain information on the chromatin landscape at sites bound by EBNA 2 and the EBNA 3 proteins, we examined histone modifications mapped by ENCODE in the EBV-immortalised LCL GM12878 at the locations of the top 1000 most significant EBNA binding sites mapped in Mutu III BL cells. The GM12878 LCL expresses the same set of EBV latent genes expressed in Mutu III cells (Figure S1) and although there may be some variation in chromatin landscape between BL cells and LCLs, this LCL represents a useful proxy. We found that the majority of EBNA 2 binding sites could be broadly grouped into three clusters defined by high levels of acetylated lysine 27 on Histone H3 (H3K27ac) and the presence of mono-methylated lysine 4 on Histone H3 (H3K4me1), a profile indicative of active enhancers [63] (Figure 2A). The remaining EBNA 2 binding sites displayed the characteristic H3K4me1 enhancer mark, but lacked significant levels of H3K27ac, a profile indicative of poised/inactive enhancers [64] (Figure 2A). These poised EBNA 2 enhancer sites lacked detectable levels of H3K27me3 that can be found in association with some classes of poised enhancers [65]. Plotting average histone modification ChIP-seq signals across the top 1000 EBNA 2 binding sites confirmed enrichment of H3K4me1 and H3K27ac and the absence of H3K27me3 (Figure S3).

A proportion of the top 1000 EBNA 3 binding sites in the human genome also displayed the characteristics of active enhancers (H3K4me1+, H3K27ac+) (Figure 2B). It is noteworthy that these active EBNA 3 enhancer sites were also associated with high-levels of EBNA 2 binding (Figure 2B). The remaining EBNA 3 binding sites displayed the characteristics of poised enhancers (H3K4me1+, H3K27ac−) (Figure 2B). Plotting average histone modification ChIP-seq signals across the top 1000 EBNA 3 binding sites confirmed enrichment of H3K4me1 and H3K27ac and the absence of H3K27me3, similar to that observed for EBNA 2 binding sites (Figure S4). The chromatin landscape at EBNA 3 binding sites is consistent with the function of these proteins as both repressors and activators of transcription, but it is interesting that our analysis did not detect any colocalization of the H3K27me3 mark, or the K27 methyltransferase EZH2 that has been associated with EBNA 3-mediated gene repression [37], [48], [53], [55] (Figure 2B and S4). However, H3K27me3 is often broadly spread over promoter and gene bodies and the lack of H3K27me3 at EBNA 3 binding sites may be consistent with our recent observation that the repression of *ADAM 28* and *ADAMDEC1* by EBNA 3C via an intergenic enhancer was associated with larger increases in H3K27me3 within the genes than at the actual binding site [37].

### EBNA 2 and 3 binding sites are also bound by B-cell specific transcription factors

To determine whether specific cellular transcription factor binding motifs were enriched at sites bound by EBNA 2, 3A, 3B and 3C we performed unbiased motif searching using the top 300 most significant EBNA binding sites. We found that EBNA 2 sites were enriched for RBP-Jκ and EBF1 motifs and that binding sites for the EBNA 3s were enriched for PU.1 motifs (Figure 2C and D). All shared sites were enriched for PU.1 and EBF1 motifs (Figure 2E). These data provide support for a role for RBP-Jκ and PU.1 in EBNA 2 and EBNA 3C gene targeting *in vivo* and corroborate recent reports of enrichment of EBF1 motifs at EBNA 2 binding sites in LCLs, implicating EBF1 in EBNA 2 targeting or binding stabilization [12]–[15], [17], [42]–[45].

We next examined transcription factor binding at EBNA 2 and EBNA 3 binding sites using recently published RBP-Jκ ChIP-seq data from the EBV-immortalised LCL, IB4 [17] and ENCODE GM12878 ChIP-seq data available for numerous transcription factors and regulators. As expected, the top 1000 EBNA 2 binding sites mapped in Mutu III cells were also extensively bound by both EBNA 2 and the EBNA 2 targeting partner RBP-Jκ in IB4 cells. EBNA 2 binding sites were however also bound by a plethora of transcription factors expressed in B cells that have not been previously implicated in EBNA 2 targeting, including BATF, Bcl11a, Bcl3, IRF4, PAX5, SP1 and TCF12 (Figure S5). Binding of the histone acetyl transferase p300, a co-activator characteristically associated with enhancers, is also evident at EBNA 2 sites. Plotting average ChIP-seq signals across EBNA 2 binding sites confirmed the binding of BATF, Bcl11a, Bcl3, IRF4, PAX5, SP1, TCF12 and p300 (Figure S6). These plots also detected association of the EBNA 2 binding protein PU.1 [15] with EBNA 2 binding sites. Interestingly, EBF1 ChIP-seq signals were high across EBNA 2 binding sites (Figure S6) consistent with the presence of EBF1 binding motifs at EBNA 2 sites (Figure 2C and E) and recent studies implicating EBF1 in EBNA 2 transcriptional regulation [17]. Our analysis revealed that the top 1000 EBNA 3 binding sites were also extensively bound by the same set of factors bound at EBNA 2 sites including RBP-Jκ, BATF, Bcl11a, Bcl3, IRF4, PAX5, SP1, TCF12, PU.1, EBF1 and p300 (Figures S5 and S7).

In summary, our co-association analyses detect the binding of multiple transcription factors at EBNA binding sites, with the binding of known targeting partners indistinguishable from that of a plethora of other transcription factors. These results are consistent with genome-wide ChIP-seq analyses by ENCODE that detect extensive context and cell-type specific co-association of multiple transcription factors with the same regulatory sites [66]. This is likely to reflect the accessibility of open chromatin at the binding site and protein-protein interactions between transcription factors. As a result, although our motif searching confirmed a potential role for EBF1 in EBNA 2 targeting, examination of the binding of particular transcription factors at EBNA binding sites through co-association analysis does not appear to be useful for the identification of the specific factors that may involved in targeting or stabilising the binding of EBNA 2 and 3 proteins to cellular DNA regulatory elements.

### Investigating the role of co-incident EBNA 2 and 3 protein binding at key gene loci

Since our analysis of EBNA 2 and 3 binding had revealed a large number of shared binding sites, we set out to investigate the nature of this shared binding and its role in the control of epigenetic reprogramming by EBV. We carried out follow-up studies on three cellular genes (*CTBP2, WEE1* and *ITGAL*) with highly significant common binding sites for EBNA 2 and EBNA 3 proteins with ChIP-seq peak heights of >10 sequence reads per million background subtracted reads. These genes are involved in regulating key processes relevant to cellular transformation including transcription repression, cell-cycle and cell adhesion and activation. C-terminal binding proteins (CtBPs) encoded by the closely-related genes *CTBP2* and *CTBP1* mediate transcriptional repression of a number of key tumour suppressor genes [67]. CtBPs play a key role in EBV transformation; CtBP binding by EBNA 3A and EBNA 3C is required for the epigenetic repression of p16^INK4a^ in infected cells [53]. *WEE1* encodes a cell-cycle kinase and negative regulator of CDK1 that controls the transition from G2 into mitosis, a process that is deregulated at multiple potential levels by EBV [68]–[70]. The B-cell adhesion protein integrin alpha L encoded by the *ITGAL* gene forms part of the heterodimeric activation antigen, LFA-1, that is upregulated in EBV-infected cells [71]. These three genes also provide representative examples of co-incident binding sites at intragenic (*CTBP2*) and downstream (*WEE1*) long-range locations and at a gene promoter (*ITGAL*). *CTBP2* and *WEE1* represent novel EBV cellular gene targets identified in our study as the closest genes to EBNA 2 and 3 binding sites; *ITGAL* was previously identified as a gene repressed by EBNA 3B and 3C by White *et al* in microarray studies of BL cells infected with recombinant knock-out EBVs [38]. Expression of the LFA-1 heterodimer composed of integrin alpha L (CD11a) and beta 2 integrin has also been shown to be upregulated by the EBV membrane protein, LMP1 [72].

### EBNA 3A, 3B and 3C bind the C-terminal binding protein 2 (*CTBP2*) enhancer

**Figure 3:**
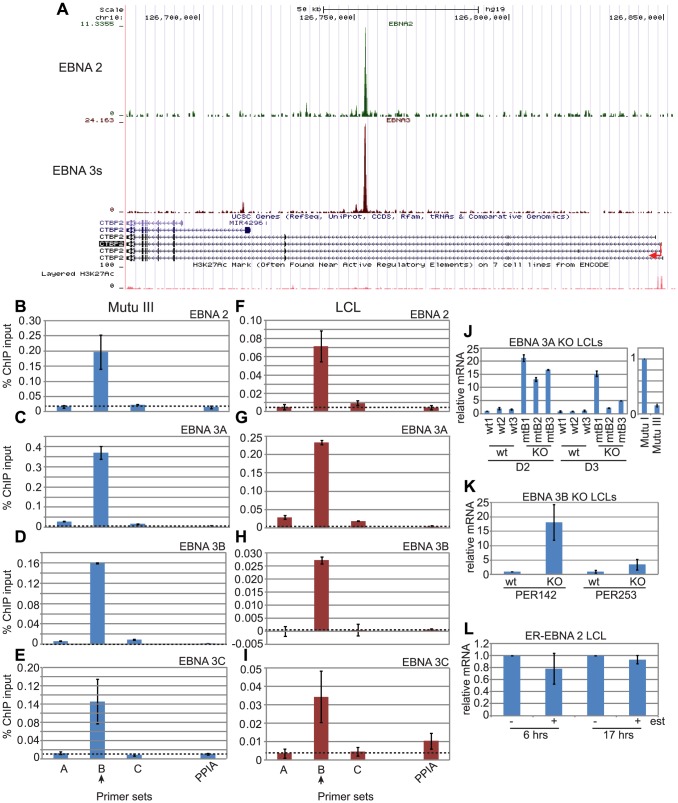
EBNA 2, 3A, 3B and 3C binding at the *CTBP2* locus in EBV-infected cells. (A) The number of EBNA 2 (green) and EBNA 3 (red) sequencing reads from immunoprecipitated Mutu III DNA are plotted per million background-subtracted total reads and aligned with the human genome. The direction of gene transcription is indicated by the red arrow. GM12878 LCL H3K27ac ChIP-seq data from ENCODE are shown at the bottom of the panel. Panels B-E show ChIP-QPCR carried out in Mutu III cells and panels F-I show data from the PER253 B95.8 LCL. Precipitated DNA was analysed using primer sets located at the binding site (set B) or regions on either side of the binding site (sets A and C). Primers spanning the transcription start site of the cellular gene encoding peptidylprolyl isomerase A (PPIA) that is not regulated or bound by the EBNAs provide a background binding control (indicated by dotted lines). (B) and (F) ChIP using anti-EBNA 2 antibodies. (C) and (G) ChIP using anti-EBNA 3A antibodies. (D) and (H) ChIP using anti-EBNA 3B antibodies. (E) and (I) ChIP using anti-EBNA 3C antibodies. Percentage input signals, after subtraction of no antibody controls, are shown as the mean +/− range of two independent experiments. (J) Q-PCR analysis of *CTBP2* transcript levels using cDNA from wild-type LCLs (wt1, 2 and 3) and LCLs established from EBNA 3A knock-out viruses (mtB1, B2 and B3) in two different donor backgrounds (D2 and D3). Transcript levels were normalised to GAPDH levels and expressed relative to the level in D2 wt1 cells. (K) Q-PCR analysis of *CTBP2* transcript levels using cDNA from wild-type LCLs infected with B95.8 virus (wt) and EBNA 3B knock-out LCLs (KO) in two different donor backgrounds (PER142 and PER253). Transcript levels were normalised to GAPDH levels and expressed relative to the level in wt cells for each donor. (L) Q-PCR analysis of *CTBP2* transcript levels using cDNA from the ER/EB 2.5 LCL expressing EBNA 2 that is active in the presence of β-estradiol (+ est) and inactive in the absence of β-estradiol (−est). Cells were incubated in β-estradiol-free media for 4 days prior to re-addition of β-estradiol or DMSO control for 6 or 17 hrs. Transcript levels were normalised to GAPDH levels and expressed relative to the level in the absence of β-estradiol for each time course. All cDNA results (J–L) show the mean −/+ range of two independent QPCR reactions each performed in duplicate.

A single co-incident binding site for EBNA 2 and EBNA 3 proteins was mapped in the second intron of *CTBP2*, 95 kb downstream from the TSS (Figure 3). To confirm our ChIP-seq data we carried out ChIP-QPCR using EBNA 2-specific antibodies in the Mutu III cells used for ChIP-seq analysis using sets of primers designed to amplify the binding site, adjacent control regions and the peptidylprolyl isomerase 1 (PPIA) control gene, which is not bound or regulated by the EBNAs. This analysis confirmed that EBNA 2 bound specifically to the *CTBP2* intragenic site *in vivo* (Figure 3B). Since our ChIP-seq analysis was carried out using an antibody that precipitated all three EBNA 3 proteins, we also carried out ChIP-QPCR in Mutu III cells to examine the binding of EBNA 3A, 3B and 3C proteins individually using anti-EBNA 3A, anti-EBNA 3B and anti-EBNA 3C antibodies that we verified do not cross-react with other family members (Figure S8). Our results demonstrated that EBNA 3A, 3B and 3C were all able to associate with the *CTBP2* site in Mutu III cells (Figure 3B–E). We also extended our ChIP-QPCR analysis to examine binding of EBNA 2, 3A, 3B and 3C to the *CTBP2* site in an EBV-immortalized LCL to investigate any potential cell-type specific differences in binding. As in Mutu III cells, we found that EBNA 2, 3A, 3B and 3C were all able to associate with the CTBP2 intragenic site in an LCL (Figure 3F–I).

Previous expression microarray analyses have not identified *CTBP2* as an EBNA 2, 3A, 3B or 3C-regulated gene. However, examination of microarray data from a study carried out by Hertle *et al* [32] (re-analysed by Dr R. White and colleagues and available at www.epstein-barrvirus.org.uk) using sets of LCLs infected with wild-type or EBNA 3A mutant EBV (Figure S9) revealed that expression of *CTBP2* was upregulated in LCLs infected with EBNA 3A knock-out (KO) EBV. These data implicate EBNA 3A as a repressor of *CTBP2* transcription. QPCR analysis of *CTBP2* mRNA levels in these EBNA 3A KO LCLs confirmed that loss of EBNA 3A leads to a dramatic upregulation of *CTBP2* transcript levels (Figure 3J). The predominant isoform of *CTBP2* expressed in these cells initiates from the third most distal promoter (Figure S10). We also found that *CTBP2* mRNA levels were higher in a latency I BL cell line expressing only EBNA 1 (Mutu I) compared to Mutu III cells, the latency III derivative of this cell line that expresses all EBV latent proteins (Figure 3J). To investigate the specific effects of EBNA 3B on *CTBP2* mRNA expression we analysed pairs of LCLs infected with wild-type or EBNA 3B KO EBV (Figure S9). Consistent with our observations in EBNA 3A KO LCLs, we found that a lack of EBNA 3B expression also lead to an increase in *CTBP2* transcript levels (Figure 3K). Taken together these data strongly implicate EBNA 3A and 3B binding at the *CTBP2* locus in transcriptional repression of this gene. Interestingly, during our analysis we found that *CTBP2* expression was cell-line specific; significant levels of *CTBP2* transcripts were not detectable in a number of cell lines previously used in expression microarray studies by White *et al* [38]. These include a set of wild-type and EBNA 3B knock-out LCLs and the BL31 and BL2 cells used to generate a series of EBNA 3A, 3B or 3C knock-out cell lines [38], [54]. As a result we have been unable to analyse the effects of EBNA 3C gene knock-out on *CTBP2* mRNA levels as the currently available EBNA 3C knock-out cell lines were generated in the BL31 or BL2 background [54].

To investigate any potential effects of EBNA 2 on *CTBP2* expression that may not have been detected in previous expression studies, we examined *CTBP2* mRNA levels in LCLs expressing a conditionally active estrogen receptor-EBNA 2 fusion protein (ER/EB 2.5) [3]. Our analysis did not detect any significant effect of the loss of EBNA 2 function on *CTBP2* transcript levels (Figure 3L). Our data therefore correlate EBNA 3A and 3B binding at the *CTBP2* site with the repression of *CTBP2* transcription by these factors, but do not detect any effect of loss of EBNA 2 on *CTBP2* mRNA levels in the presence of the EBNA 3s.

### EBNA 3 proteins repress *CTBP2* transcription by preventing enhancer-promoter looping

**Figure 4:**
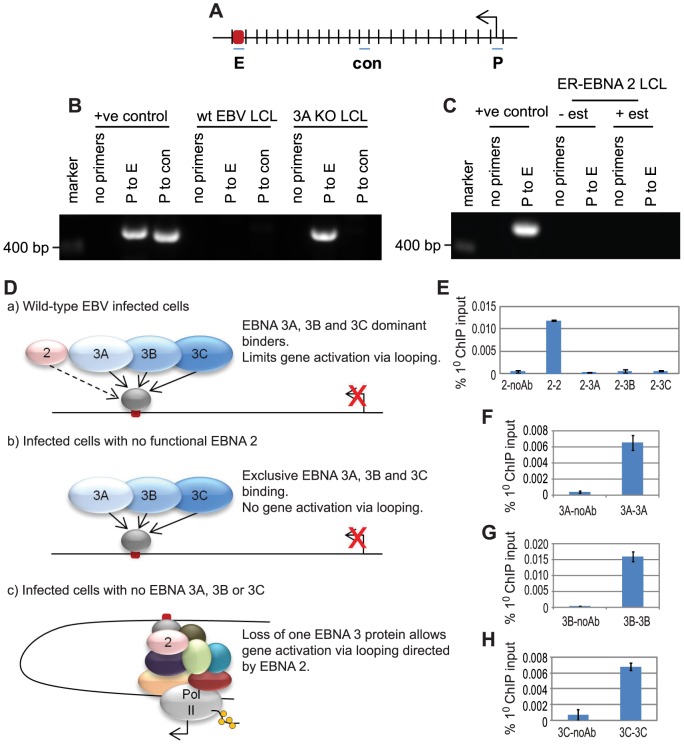
The influence of EBNA 2 and 3A on chromosome looping at the *CTBP2* locus. (A) Diagram (not to scale) showing the *Eco*R1 restriction fragments at the *CTBP2* locus that encompass the promoter (P), enhancer (E) and an intervening control region (con). The arrow indicates the direction of transcription. (B) Chromosome conformation analysis in LCLs infected with wild-type or EBNA 3A knock-out EBV using primer pairs that amplify across promoter-enhancer or promoter-control ligation junctions. Positive controls show PCR amplification from control digestion and ligation reactions carried out using PCR-amplified DNA fragments encompassing the promoter, enhancer and control regions. (C). Chromosome conformation capture analysis in the ER-EB 2.5 LCL expressing EBNA 2 that is active in the presence of β-estradiol (+ est) and inactive in the absence of β-estradiol (−est). (D) Model for the control of chromatin looping by EBNA 2 and 3 proteins at *CTBP2*. (E) Re-ChIP analysis using anti-EBNA 2 antibodies in the first round of ChIP followed by a second round of ChIP in absence of antibody or using anti-EBNA 2, EBNA 3A, EBNA 3B or EBNA 3C antibodies. Results show mean percentage primary input −/+ range of two independent Q-PCR reactions from a representative experiment. (F) Control re-ChIP analysis using anti-EBNA 3A antibodies in the first round followed by re-precipitation in the absence of antibody or using anti-EBNA 3A antibodies. (G) Control re-ChIP analysis using anti-EBNA 3B antibodies in the first round followed by re-precipitation in the absence of antibody or using anti-EBNA 3B antibodies. (H) Control re-ChIP analysis using anti-EBNA 3C antibodies in the first round followed by re-precipitation in the absence of antibody or using anti-EBNA 3C antibodies.

To determine the role of the long-range *CTBP2* intragenic EBNA 2, 3A, 3B and 3C binding site in controlling *CTBP2* transcription, we performed chromosome conformation capture analysis [73] to examine potential interactions between this site and the *CTBP2* promoter through the formation of chromatin loops. Following digestion of cross-linked chromatin using an enzyme that cleaves intervening DNA, enhancer and promoter regions can be ligated together at low dilution using this technique only if these elements are in close spatial proximity (Figure 4A). We found that PCR amplification products using primers spanning the *CTBP2* enhancer-promoter ligation junction could not be detected in LCLs generated from wild-type EBV where all EBNAs are expressed, but were readily detectable in EBNA 3A knock-out LCLs (Figure 4B). These data indicate that enhancer-promoter chromatin loops form in the absence of EBNA 3A but not in wild-type infected cells. Loop formation correlated with increased *CTBP2* transcription in EBNA 3A knock-out LCLs indicating that looping at the *CTBP2* locus is associated with active transcription (Figure 3J). These data are consistent with a model where EBNA 3A represses *CTBP2* transcription by preventing the formation of chromatin loops that mediate gene activation. Concordantly, chromosome conformation capture experiments in LCLs expressing a conditionally active ER-EBNA 2 demonstrated that *CTBP2* promoter-enhancer interactions were absent in these cells irrespective of whether EBNA 2 was functional (+/− β-estradiol) (Figure 4C and D) presumably as a result of the dominant binding and prevention of looping by the EBNA 3 proteins.

To directly examine whether EBNA 2 and 3 proteins can associate with the *CTBP2* enhancer at the same time or compete for binding, we carried out re-ChIP analysis. Primary precipitation of chromatin was carried out using an EBNA 2-specific antibody and immunoprecipitated protein-DNA complexes were then eluted from the bead matrix and a second round of precipitation carried out using EBNA 2, 3A, 3B or 3C-specific antibodies (Figure 4E). Our analysis detected re-precipitation of EBNA 2 bound to the *CTBP2* enhancer using EBNA 2-specific antibodies as expected, but EBNA 3A, 3B and 3C were not precipitated from EBNA 2-DNA complexes, despite the fact that they can be re-precipitated with the antibodies used (Figure 4F–H). Reciprocal re-ChIP experiments carried out using EBNA 3A, 3B or 3C antibodies in the first round and EBNA 2 antibodies in the second round confirmed that EBNA 2 could not be precipitated from EBNA 3-DNA complexes (Figure S11). Our results therefore indicate that EBNA 3A, 3B and 3C do not bind the *CTBP2* enhancer at the same time as EBNA 2. Binding profiles obtained in ChIP therefore represent independent binding of EBNA 2 or EBNA 3 proteins to this site in different cells within the total cell population assayed. In summary, our results are consistent with *CTBP2* transcriptional repression by EBNA 3 proteins via competitive binding at the *CTBP2* intragenic enhancer (Figure 4D).

### Cell-type specific differential binding of EBNA 3 proteins to two downstream distal enhancers at the *WEE1* locus

**Figure 5:**
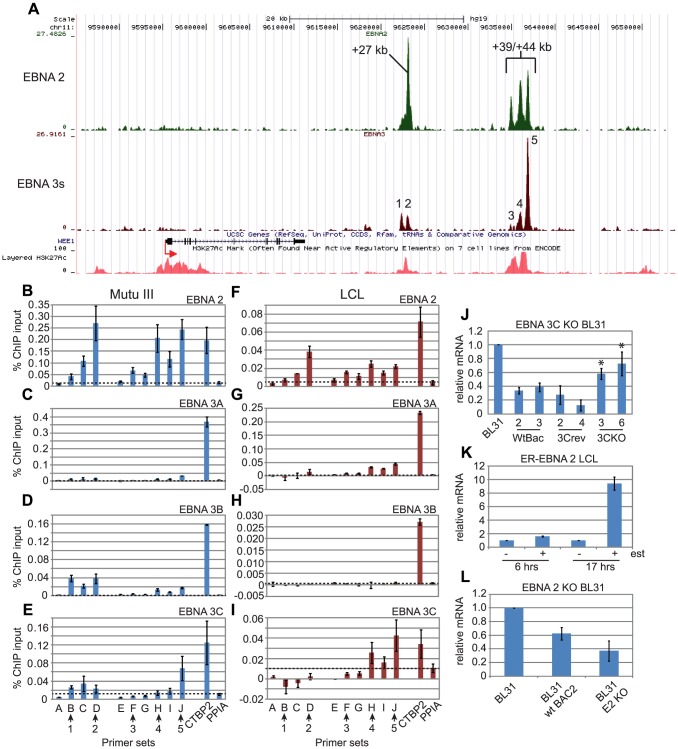
EBNA 2 and EBNA 3 protein binding at the *WEE1* locus in EBV infected cells. (A) EBNA 2 (green) and EBNA 3 (red) sequencing reads at the *WEE1* locus (displayed as described in Figure 3). Panels B–E show ChIP-QPCR carried out in Mutu III cells and panels F–I show data from the PER253 B95.8 LCL. Precipitated DNA was analysed using primer sets located at the binding sites (sets B, D, F, H and J) or regions adjacent to the binding sites (sets A, C, E, G and I). Binding signals at the *CTBP2* binding site in the same ChIP experiments are shown as a positive control and primers spanning the transcription start site of the cellular gene PPIA provide a background binding control (indicated by dotted lines). (B) and (F) ChIP using anti-EBNA 2 antibodies. (C) and (G) ChIP using anti-EBNA 3A antibodies. (D) and (H) ChIP using anti-EBNA 3B antibodies. (E) and (I) ChIP using anti-EBNA 3C antibodies. Percentage input signals, after subtraction of no antibody controls, are shown as the mean −/+ range of two independent ChIP experiments. (J) Q-PCR analysis of *WEE1* transcript levels using cDNA from BL31 parental cells and BL31 cells infected with wild-type recombinant EBV (wtBac-2 and 3), EBNA 3C knock-out EBV (3C KO-3 and 6) or EBNA 3C revertant EBV (3Crev-2 and 4). Transcript levels were normalised to GAPDH levels and expressed relative to the level in parental BL31 cells. * indicates a p-value of <0.01 (students t-test) compared to the wtBac-2 cell line. (K) Q-PCR analysis of *WEE1* transcript levels using cDNA from the ER/EB 2.5 LCL expressing EBNA 2 that is active in the presence of β-estradiol (+ est) and inactive in the absence of β-estradiol (−est). Cells were incubated in β-estradiol-free media for 4 days prior to re-addition of β-estradiol for 6 or 17 hrs. Transcript levels were normalised to GAPDH levels and expressed relative to the level in the absence of β-estradiol. All cDNA results (J–L) show the mean −/+ standard deviation of three independent QPCR analyses from two independent cDNA preparations.

ChIP-seq detected binding of EBNA 2 and 3 proteins to co-incident binding sites located in two clusters +27 kb and +39 to 44 kb downstream from the *WEE1* TSS (Figure 5). The first cluster, enhancer 1 (+27 kb) has two distinct binding sites for EBNA 3 proteins (sites 1 and 2), with EBNA 2 binding detected predominantly at the most distal of these two sites (site 2). The second cluster, enhancer 2 (+39 to 44 kb) contains 3 binding sites (sites 3–5), with the highest binding signal for EBNA 3 proteins detected at the most distal site (site 5). Both clusters of EBNA 2 and 3 binding sites coincide with peaks of H3K27ac detected in ENCODE ChIP-seq experiments in the GM12878 LCL indicating that the binding sites are located within active gene regulatory regions (Figure 5).

ChIP-QPCR analysis confirmed EBNA 2 binding to all five sites in both Mutu III cells and an LCL (Figure 5). Consistent with ChIP-seq data, we detected the highest levels of EBNA 2 binding at site 2 in both cell lines (Figure 5B and F). EBNA 3A and 3B binding at *WEE1* was low-level, with only small amounts of EBNA 3A binding at sites 4 and 5 in LCLs and weak EBNA 3B binding at sites 1 and 2 in Mutu III cells (Figure 5C, D, G and H). In contrast, EBNA 3C bound specific *WEE1* enhancer sites at levels similar to that detected at *CTBP2*, although there were cell-type specific differences in the binding profile (Figure 5E and I). EBNA 3C bound predominantly at site 5 in Mutu III cells, but at both sites 4 and 5 in an LCL (Figure 5E and I).

Like *CTBP2*, *WEE1* had not previously been documented as a gene regulated by EBNA 2 or EBNA 3 proteins. However, examination of microarray data obtained by White *et al* [38] from BL31 cells infected with a series of EBNA 3 knock-out viruses provided evidence of repressive effects of EBNA 3C (but not EBNA 3A and 3B) on *WEE1* mRNA expression (www.epstein-barrvirus.org.uk). QPCR analysis of these BL31 cell lines confirmed that *WEE1* transcript levels were modestly but significantly increased in cells infected with EBNA 3C knock-out EBV compared to cells infected with wild-type EBV or revertant viruses (Figure 5J). Examination of microarray data obtained by White *et al* [38] and Hertle *et al* [32] using EBNA 3B and EBNA 3A knock-out LCLs respectively, confirmed that EBNA 3A and EBNA 3B had no detectable effect on *WEE1* mRNA levels. Our data are therefore consistent with EBNA 3C binding to the most distal enhancer region (enhancer 2) downstream of *WEE1* playing a dominant role in the repression of *WEE1* transcription. The low-level association of EBNA 3A and 3B at *WEE1* enhancer sites however, does not detectably affect *WEE1* mRNA levels in BL cells or LCLs.

To address the potential contribution of EBNA 2 in the regulation of *WEE1* transcription we examined *WEE1* mRNA expression in BL31 cells infected with an EBNA 2 knock-out virus and in LCLs expressing conditionally active EBNA 2. Our analysis revealed that *WEE1* mRNA levels are reduced in the absence of EBNA 2 activity, implicating EBNA 2 in the positive regulation of *WEE1* transcription (Figure 5K and L). Taken together our data indicate that the level of *WEE1* transcription in EBV-infected cells is likely to be determined by a balance between gene repression by EBNA 3C that is counteracted by EBNA 2.

### EBNA 3C represses *WEE1* transcription by directing the formation of enhancer-promoter loops

**Figure 6:**
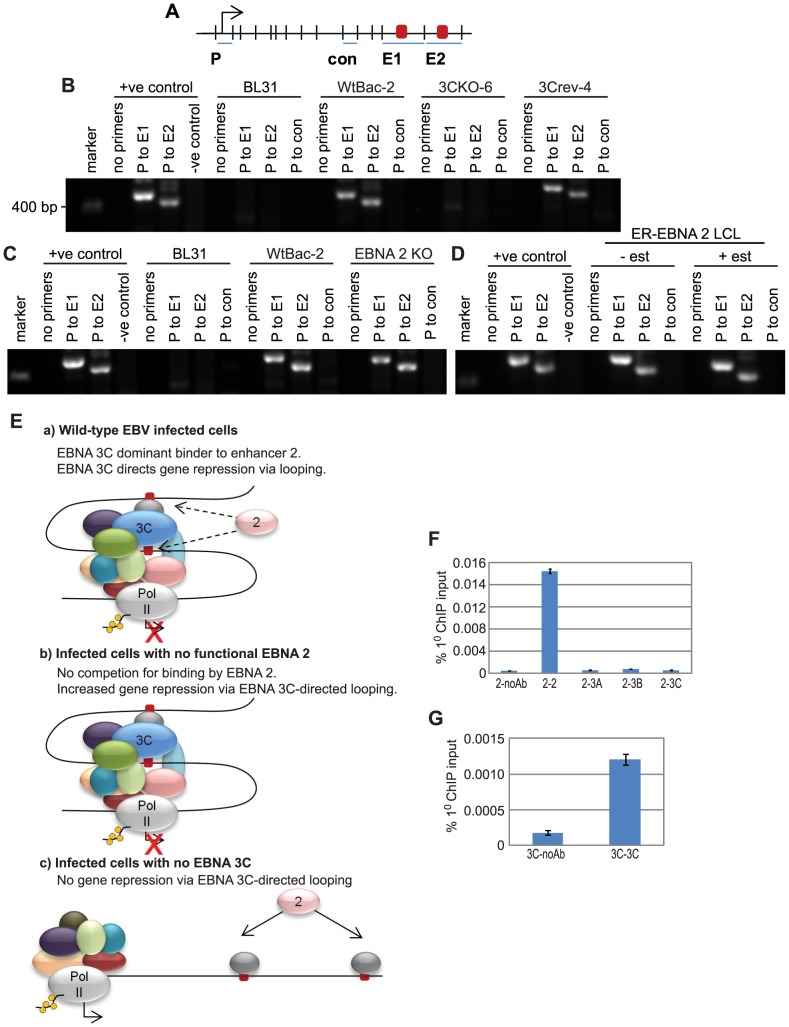
The influence of EBNA 2 and 3C on chromosome looping at the *WEE1* locus. (A) Diagram (not to scale) showing the *Eco*R1 restriction fragments at the *WEE1* locus that encompass the promoter (P), two downstream enhancers (E1 and E2) and an intervening control region (con). The arrow indicates the direction of transcription. (B) Chromosome conformation analysis in BL31 parental cells and BL31 cells infected with wild-type recombinant EBV (wtBac-2), EBNA 3C knock-out EBV (3CKO-3) or EBNA 3C revertant EBV (3Crev-4) using primer pairs that amplify across promoter-enhancer or promoter-control ligation junctions. Positive controls show PCR amplification from control digestion and ligation reactions carried out using PCR-amplified DNA fragments encompassing the promoter and enhancers. (C) Chromosome conformation analysis in BL31 parental cells and BL31 cells infected with wild-type recombinant EBV (wtBac-2) or EBNA 2 KO EBV. (D) Chromosome conformation capture analysis in the ER-EB 2.5 LCL expressing EBNA 2 that is active in the presence of β-estradiol (+ est) and inactive in the absence of β-estradiol (−est). (E) Model for the control of chromatin looping by EBNA 2 and 3 proteins at *WEE1*. (F) Re-ChIP analysis in Mutu III cells using anti-EBNA 2 antibodies in the first round of ChIP followed by a second round of ChIP in absence of antibody or using anti-EBNA 2 or EBNA 3A, 3B or 3C antibodies. Primers at peak 5 in enhancer 2 were used for analysis. Results show mean percentage primary input −/+ range of two independent Q-PCR reactions from a representative experiment. (G) Control re-ChIP analysis using anti-EBNA 3C antibodies in the first round followed by re-precipitation in the absence of antibody or using anti-EBNA 3C antibodies.

To examine potential looping interactions between the downstream *WEE1* enhancers and the *WEE1* promoter and their regulation by EBNA 2 and 3C, we performed chromosome conformation capture analysis. In contrast to the situation at the *CTBP2* locus, our results indicated that looping interactions at *WEE1* were associated with transcriptional repression. Looping between both enhancer 1 (sites 1 and 2) and enhancer 2 (sites 3, 4 and 5) and the promoter was not detectable in parental EBV negative BL31 cells, but was detectable in wild-type EBV infected BL31 cells where *WEE1* expression is repressed (Figure 5J). Consistent with the key role played by EBNA 3C in transcriptional repression of *WEE1*, looping interactions were absent in BL31 cells infected with EBNA 3C knock-out viruses indicating that EBNA 3C is required to maintain enhancer-promoter looping (Figure 6B).

The dominant role of EBNA 3C binding in mediating transcriptional repression of *WEE1* via chromatin looping was supported by our observations that looping interactions were maintained in BL31 cells infected with EBNA 2 knock-out viruses and in an ER-EBNA 2 LCL when EBNA 2 function was inhibited through β-estradiol withdrawal (Figure 6C and D). Taken together these data support a model where EBNA 3C binding to a downstream enhancer element directs chromatin looping with the *WEE1* promoter to facilitate transcriptional repression (Figure 6E). Given that loss of EBNA 2 decreases *WEE1* transcription (Figure 5J), EBNA 2 may compete with EBNA 3C for binding to *WEE1* enhancer sites and limit EBNA 3C-mediated repression in infected cells. It is likely that the semi-quantitative chromosome conformation capture assay is not able to detect the increased looping that may occur in the absence of EBNA 2. Consistent with competitive binding of EBNA 2 and 3 proteins and our observations at the *CTBP2* locus, re-ChIP experiments did not detect co-association of EBNA 2 and 3C at *WEE1* enhancer 2 (Figure 6F and G and S11).

### Cell-type specific differential binding of EBNA 3 proteins to the *ITGAL* promoter

**Figure 7:**
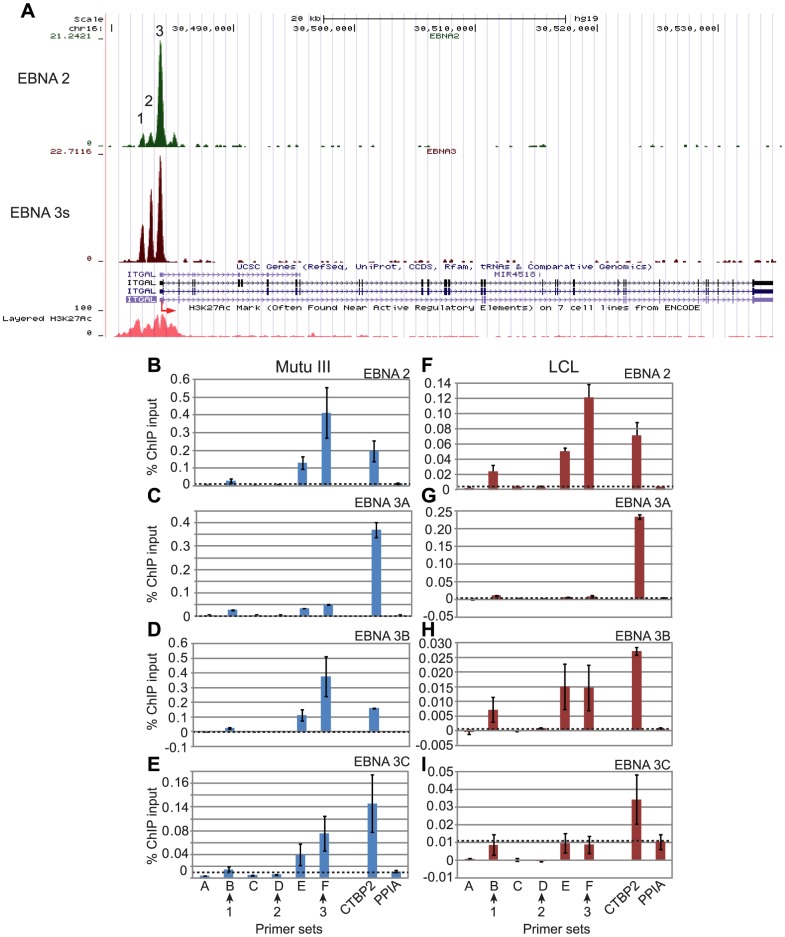
EBNA 2 and EBNA 3 protein binding at the *ITGAL* promoter in EBV-infected cells. (A) EBNA 2 (green) and EBNA 3 (red) sequencing reads from immunoprecipitated Mutu III DNA plotted as in Figure 3. Panels B–E show ChIP-QPCR carried out in Mutu III cells and panels F–I show data from the PER253 B95.8 LCL. Precipitated DNA was analysed using primer sets located at the binding sites (sets B, D, and F) or regions adjacent to the binding sites (sets A, C, and E). Binding signals at the *CTBP2* binding site in the same ChIP experiments are shown as a positive control and primers spanning the transcription start site of the cellular gene PPIA provide a background binding control (indicated by dotted lines). (B) and (F) ChIP using anti-EBNA 2 antibodies. (C) and (G) ChIP using anti-EBNA 3A antibodies. (D) and (H) ChIP using anti-EBNA 3B antibodies. (E) and (I) ChIP using anti-EBNA 3C antibodies. Percentage input signals, after subtraction of no antibody controls, are shown as the mean −/+ range of two independent ChIP experiments.

**Figure 8:**
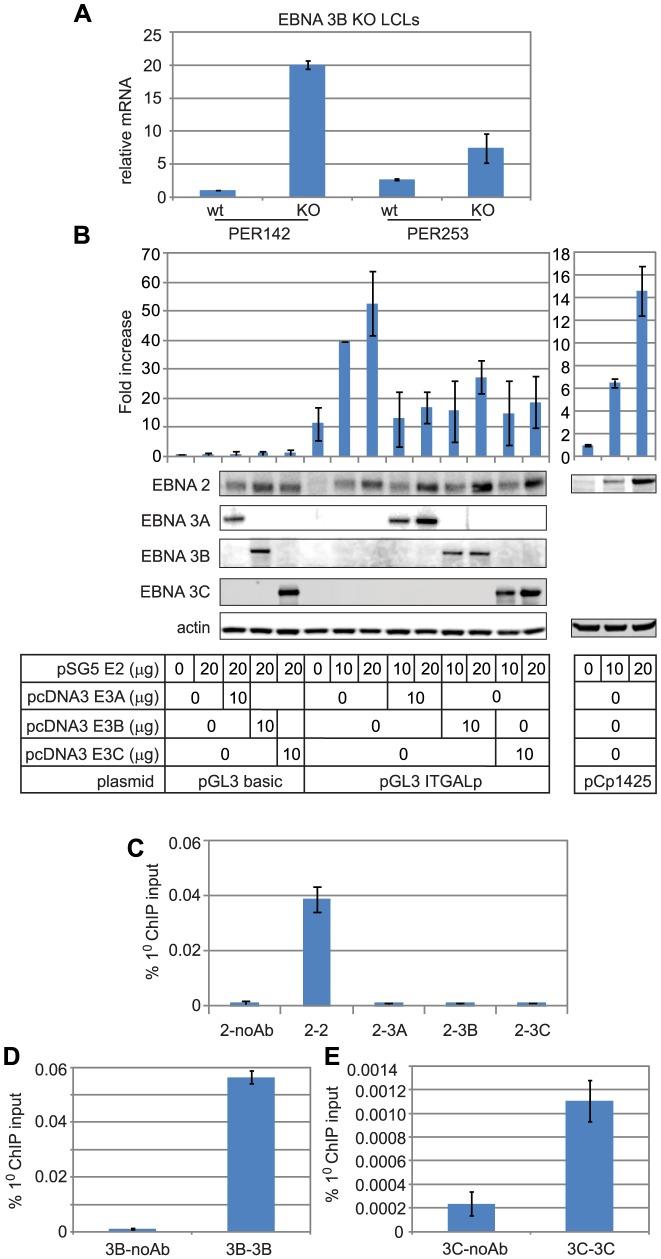
The Effect of EBNA 2 and 3 proteins on *ITGAL* expression. (A) Q-PCR analysis of *ITGAL* transcript levels using cDNA from wild-type LCLs infected with B95.8 virus (wt) and EBNA 3B knock-out LCLs (KO) in two different donor backgrounds (PER142 and PER253). Transcript levels were normalised to GAPDH levels and expressed relative to the level in wt cells for each donor. Results show the mean −/+ range of two independent QPCR reactions each performed in duplicate. (B) Luciferase reporter assays carried out in DG75 cells transiently transfected with 2 µg of the control vector pGL3 basic, an *ITGAL* promoter-luciferase reporter (pGL3 *ITGAL*p) or the EBV C promoter reporter (pCp1425GL2) (right panel) in the absence or presence of 10 or 20 µg of EBNA 2, 3A, 3B or 3C expressing constructs. Firefly luciferase signals were normalised to Renilla luciferase signals from the co-transfected control plasmid pRL-TK (1 µg). Results show the mean −/+ standard deviation of 3 independent experiments and are expressed relative to the pGL3 basic signal (left panel) or the Cp1425GL2 signal (right panel) in the absence of EBNA 2. Western blot analysis of EBNA 2, 3A, 3B and 3C expression levels in transfected cells. Each set of blots was also probed for actin as a loading control. (C) Re-ChIP analysis in Mutu III cells using anti-EBNA 2 antibodies in the first round of ChIP followed by a second round of ChIP in absence of antibody or using anti-EBNA 2, EBNA 3B or EBNA 3C antibodies. Primers at peak 3 were used for analysis. Results show mean percentage primary input −/+ range of two independent Q-PCR reactions from a representative experiment. (D) Control re-ChIP analysis using anti-EBNA 3B antibodies in the first round followed by re-precipitation in the absence of antibody or using anti-EBNA 3B antibodies. (E) Control re-ChIP analysis using anti-EBNA 3C antibodies in the first round followed by re-precipitation in the absence of antibody or using anti-EBNA 3C antibodies.

At *ITGAL* a series of three co-incident binding sites for EBNA 2 and EBNA 3 proteins are located in a 2 kb region encompassing the TSS that coincides with a peak of LCL H3K27ac (Figure 7). Consistent with ChIP-seq analysis, ChIP-QPCR experiments confirmed that EBNA 2 bound predominantly at site 3 in both BL cells and an LCL (Figure 7B and F). Interestingly, when we examined individual binding of EBNA 3 proteins to these promoter sites, we found that EBNA 3B also bound at site 3 in both BL cells and LCLs (Figure 4D and H), but there was no significant binding of EBNA 3A in either cell line, despite our ability to detect significant levels of EBNA 3A binding at the *CTBP2* locus in the same ChIP samples (Figure 7C and G). We also found that although EBNA 3C bound site 3 in Mutu III cells at levels significantly above the background level detected at a control gene, there was no significant EBNA 3C binding in LCLs (Figure 7E and I). Remarkably, the differential and cell-type specific targeting of the *ITGAL* promoter by EBNA 3 family members we detected is entirely consistent with the documented effects of individual EBNA 3 proteins on *ITGAL* expression in these different cell backgrounds. Consistent with our observations in the Mutu III BL cell line, the White *et al* BL31 study [38] detected significant and reproducible increases in *ITGAL* expression in cell lines infected with EBNA 3B or EBNA 3C knock-out EBV, but not EBNA 3A knock-out EBV, compared to cells infected with wild-type EBV. EBNA 3B knock-out BL31 cells displayed a 3.09-fold (p-value: 2.77E-05) increase in *ITGAL* expression vs wild-type infected BL31 cells and EBNA 3C knock-out BL31 cells displayed a 2.72-fold increase (p-value: 1.09E-04) [38]. Interestingly, examination of microarray data from the White *et al* [38] and Hertle *et al* [32] LCL studies (www.epstein-barrvirus.org.uk) revealed a role for EBNA 3B, but not EBNA 3A in the repression of *ITGAL* expression in LCLs. These data are entirely consistent with our ChIP QPCR analysis where binding of EBNA 3B was detected in LCLs in the absence of any significant EBNA 3A or EBNA 3C binding (Figure 7G, H and I). We confirmed the repressive effects of EBNA 3B on *ITGAL* expression in LCLs using an additional independent set of wild-type and EBNA 3B knock-out cell lines (Figure 8A). Thus *ITGAL* is an EBNA 3B and 3C-repressed gene in BL cells and an EBNA 3B only-repressed gene in LCLs. Since EBNA 3A appears to play no role in *ITGAL* regulation, importantly our data demonstrate that it is the gene and cell-type specific binding of different members of the EBNA 3 protein family that determines the requirement for each EBNA 3 protein in the regulation of specific cellular genes.

Since *ITGAL* is upregulated by EBV LMP1 [72], a direct target gene of EBNA 2 that is not expressed in the absence of EBNA 2, the effects of LMP1 and EBNA 2 on *ITGAL* expression cannot be separated using cells which lack functional EBNA 2. We therefore examined the regulation of the *ITGAL* promoter by EBNA 2 using *ITGAL* promoter-reporter constructs containing the 2 kb region encompassing all 3 binding sites. Transient transfection assays demonstrated that EBNA 2 activated the ITGAL promoter up to 5-fold (Figure 8B). The co-expression of EBNA 3A, 3B or 3C resulted in the inhibition of EBNA 2 activation (Figure 8B). These data are consistent with a model where EBNA 3 proteins can compete with EBNA 2 for binding sites in the *ITGAL* promoter to repress *ITGAL* transcription. It therefore appears that although EBNA 3A and EBNA 3C (in LCLs) do not bind the *ITGAL* promoter site significantly *in vivo*, these proteins are able to bind and compete with EBNA 2 for binding to this site when expressed at high levels in reporter assays. Competitive binding of EBNA 2 and 3 proteins at the *ITGAL* promoter was also supported by re-ChIP analysis in the Mutu III BL cell line, where we detected no simultaneous binding of EBNA 2 with EBNA 3B or EBNA 3C (Figure 8C–E and Figure S11).

### EBNA 3 proteins also differentially bind EBNA 3-only promoter and enhancer sites

**Figure 9:**
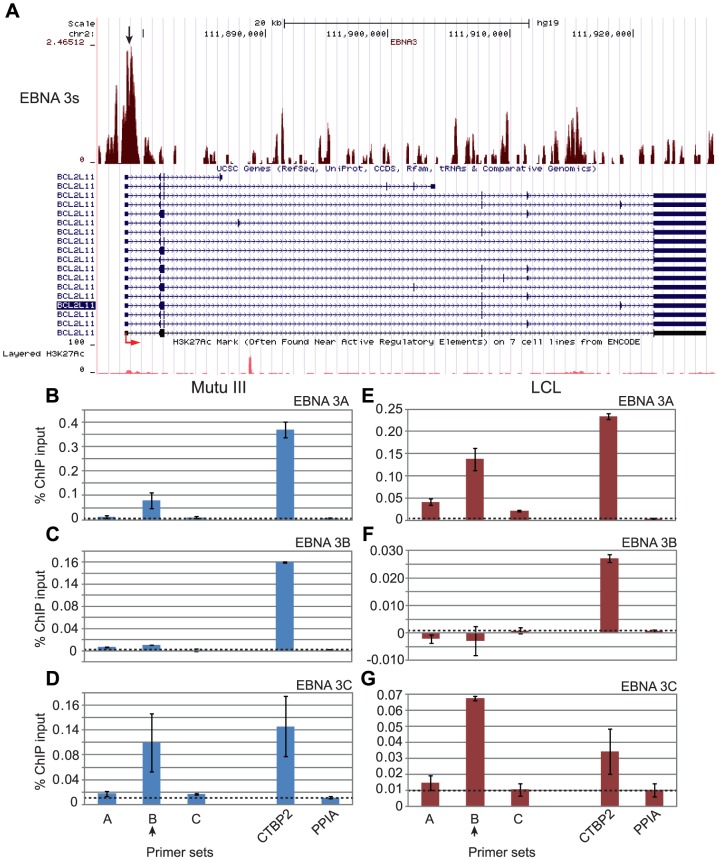
EBNA 3 protein binding at the *BCL2L11* promoter in EBV-infected cells. (A) EBNA 3 sequencing reads from immunoprecipitated Mutu III DNA plotted as in Figure 3. Panels B–D show ChIP-QPCR carried out in Mutu III cells and panels F–G show data from the PER253 B95.8 LCL. Precipitated DNA was analysed using primer sets located at the binding site (set B) or regions on either side of the binding site (sets A and C). Binding signals at the *CTBP2* binding site in the same ChIP experiments are shown as a positive control and primers spanning the transcription start site of the cellular gene PPIA provide a background binding control (indicated by dotted lines). (B) and (E) ChIP using anti-EBNA 3A antibodies. (C) and (F) ChIP using anti-EBNA 3B antibodies. (D) and (G) ChIP using anti-EBNA 3C antibodies. Percentage input signals, after subtraction of no antibody controls, are shown as the mean −/+ range of two independent experiments.

Our observations that the regulation of *ITGAL* and *WEE1* expression by members of the EBNA 3 family was directed by the differential binding of subsets of these proteins to binding sites shared by EBNA 2 led us to investigate whether this was also the case at genes regulated by EBNA 3-only sites. The *BCL2L11* (Bim) gene is known to be transcriptionally repressed by EBNA 3A and EBNA 3C, but not EBNA 3B [54] and recent studies using an epitope-tagged form of EBNA 3C have detected binding of EBNA 3C over a broad region from −3648 to +631 bps around the *BCL2L11* TSS [48]. Although the peak binding signal of this tagged EBNA 3C in BL31 cells was detected with primer sets located −247 to −147 bps upstream of the *BCL2L11* TSS, our ChIP-seq analysis fine-mapped significant EBNA 3 binding sites between +87 to +921 bps downstream of the *BCL2L11* TSS. ChIP-QPCR analysis of the binding of individual EBNA 3A, 3B and 3C proteins to this site revealed that only EBNA 3A and 3C bind at significant levels in both the Mutu III BL and an LCL, consistent with the regulation of *BCL2L11* expression by these two proteins (Figure 9B–G). We found that the peak of binding of EBNA 3A and 3C was detected by ChIP-QPCR using primer sets located +540 to +630 downstream from the TSS (set B) (Figure 9B–G).

**Figure 10:**
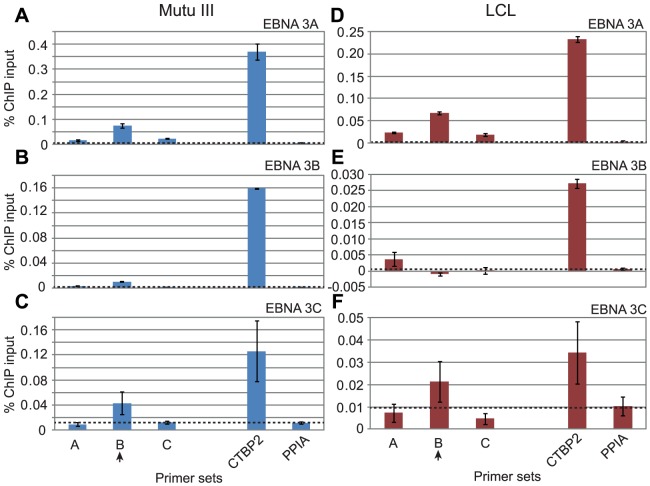
EBNA 3 protein binding at the *ADAM28/ADAMDEC1* intergenic enhancer in EBV-infected cells. ChIP-QPCR carried out in Mutu III cells (A–C) and the PER253 B95.8 LCL (D–F). Precipitated DNA was analysed using primer sets located at the centre of the binding site (set B) or the edges of the binding site (sets A and C). Binding signals at the *CTBP2* binding site in the same ChIP experiments are shown as a positive control and primers spanning the transcription start site of the cellular gene PPIA provide a background binding control (indicated by dotted lines). (A) and (D) ChIP using anti-EBNA 3A antibodies. (B) and (E) ChIP using anti-EBNA 3B antibodies. (C) and (F) ChIP using anti-EBNA 3C antibodies.

We also examined differential binding of EBNA 3A, 3B and 3C to an EBNA 3-only distal regulatory element located between the *ADAMDEC1* and *ADAM28* genes that we have previously characterised [37]. We previously demonstrated that repression of *ADAM28* and *ADAMDEC1* transcription through H3K27me3 could be mediated through the action of EBNA 3C alone and detected EBNA 3C binding to this intergenic element in EBNA 3C-expressing cells [37]. Microarray studies have also implicated EBNA 3A in *ADAMDEC1* repression in LCLs and BL cells and in *ADAM28* repression in LCLs [32], [38]. Consistent with a role for both EBNA 3A and EBNA 3C in the repression of *ADAMDEC1* transcription, we detected binding of EBNA 3A and EBNA 3C, but not EBNA 3B to the *ADAM* intergenic element in both BL cells and an LCL (Figure 10). Our data therefore indicate that the specificity of cellular gene regulation by members of the EBNA 3 family is directed by the differential binding of different family members to both unique EBNA 3 binding sites and binding sites shared with EBNA 2.

### EBNA 3C binding at the ADAM intergenic site directs transcriptional repression via chromatin looping

**Figure 11:**
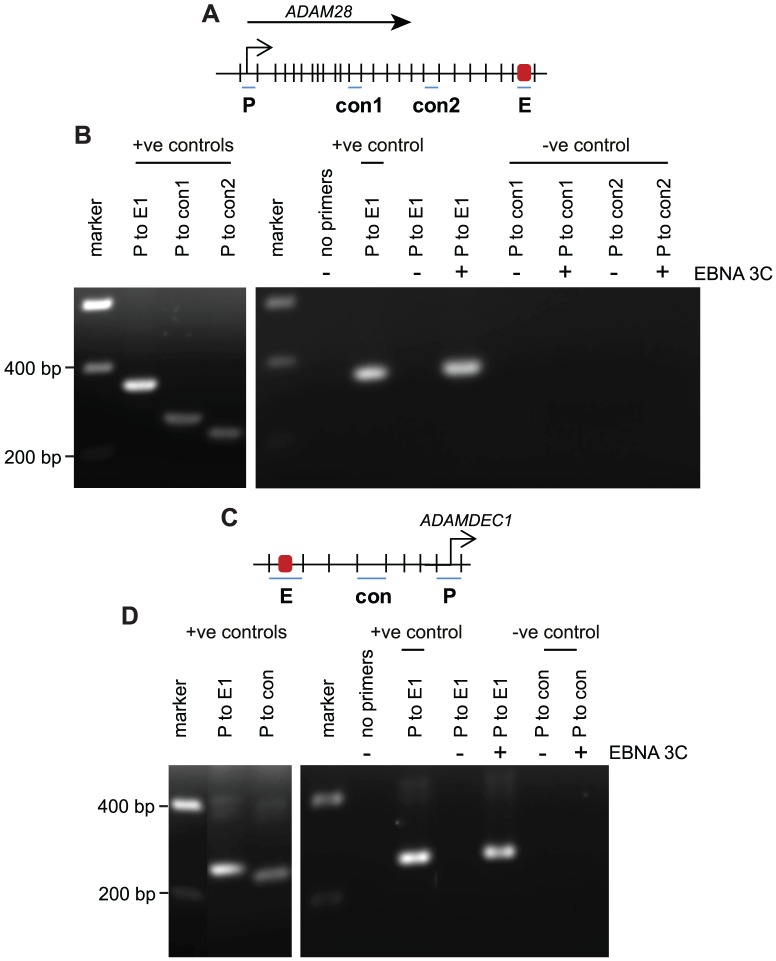
The influence of EBNA 3C on chromosome looping at the *ADAM28/ADAMDEC1* locus. (A) Diagram (not to scale) showing the *Hind*III restriction fragments around the *ADAM28* locus that encompass the promoter (P), the ADAM enhancer (E, located downstream of *ADAM28*) and two intervening control regions (con1 and con2). The arrow indicates the direction of transcription. (B) Chromosome conformation analysis of the *ADAM28* locus in the pz1 control BJAB cell line (−) and the E3C-3 stable EBNA 3C expressing cell line (+) using primer pairs that amplify across promoter-enhancer or promoter-control ligation junctions. Positive controls show PCR amplification from control digestion and ligation reactions carried out using PCR-amplified DNA fragments encompassing the promoter, enhancer and control regions. (C) Diagram (not to scale) showing the *Aci*I restriction fragments around the *ADAMDEC1* locus that encompass the promoter (P), the ADAM enhancer (E, located upstream of *ADAMDEC1*) and an intervening control region (con). The arrow indicates the direction of transcription. (D) Chromosome conformation analysis of the *ADAMDEC1* locus in the pz1 control BJAB cell line (−) and the E3C-3 stable EBNA 3C expressing cell line (+) using primer pairs that amplify across promoter-enhancer or promoter-control ligation junctions. Positive controls show PCR amplification from control digestion and ligation reactions carried out using PCR-amplified DNA fragments encompassing the promoter, enhancer and control region.

To determine whether binding of EBNA 3 proteins to unique rather than shared sites was also able to direct chromatin looping, we used chromosome conformation capture to detect potential interactions between the *ADAM* intergenic EBNA 3 binding site and both the *ADAMDEC1* and *ADAM28* promoters in an EBNA 3C-expressing B-cell line. Our analysis revealed that the intergenic enhancer was in close proximity to both the *ADAM28* promoter and the *ADAMDEC1* promoter only in cells expressing EBNA 3C (BJAB E3C-3) and not in control cells (BJAB pz1) (Figure 11). These data indicate that EBNA 3C binding represses *ADAMDEC1* and *ADAM28* transcription by promoting juxtaposition of the intergenic binding element with the two distal ADAM gene promoters to facilitate H3K27me3 via the recruitment of polycomb repressor complexes. Although our data demonstrate that EBNA 3C is able to promote chromatin looping and gene repression alone, the binding of EBNA 3A to the intergenic site and the fact that the absence of EBNA 3A expression leads to increased *ADAMDEC1* and *ADAM28* expression also implicates EBNA 3A in the repression of ADAM gene transcription.

## Discussion

The findings described here provide important mechanistic insights into two key outstanding questions in EBV biology that impact on our understanding of fundamental transcriptional control mechanisms and host-cell reprogramming by viruses (i) how are long-range cellular enhancers hijacked by EBV to direct epigenetic reprogramming? (ii) what is the basis for gene-specific and cell background specific gene deregulation by the EBNA 3 family of transcription factors? We have discovered that (i) EBV-encoded transcriptional activators and repressors modulate enhancer-promoter loop formation at cellular genes to regulate their transcription and (ii) transcriptional deregulation is controlled in a gene and cell-background specific manner through differential binding of subsets of EBV transcription factors to cellular regulatory elements. These discoveries provide important information on the mechanisms through which EBV transcriptionally and epigenetically reprogrammes the host cell. Our studies have also identified two new target genes repressed by EBNA 3 proteins in infected cells (*WEE1* and *CTBP2*) and identified additional EBV-directed mechanisms for controlling expression of the LFA-1 subunit encoded by the *ITGAL* gene. Transcriptional deregulation of these genes may play important roles in facilitating immortalization and viral persistence.

Recent studies using ChIP-seq technology have revealed that enhancer elements are more widespread than originally thought and may constitute 10% of the human genome [74]. Perturbation of enhancer function appears to play a key role in cancer development since an increasing number of single nucleotide polymorphisms at cancer risk loci map to enhancer elements and are associated with gene deregulation [75]–[78]. Our analysis has revealed that enhancer elements are also targeted and deregulated by a tumour virus to promote cellular gene deregulation during transformation, providing further evidence of a key role for the modulation of chromatin architecture during tumourigenesis.

### CTBP2

Vertebrate genomes contain two genes, *CTBP1* and *CTBP2*, that encode the closely-related proteins CtBP1 and CtBP2, collectively referred to as CtBP. CtBP was first identified as a binding partner of the adenovirus oncoprotein E1A [79]–[80] and promotes cell survival, invasion and metastatic potential through transcriptional repression of a diverse range of tumour suppressors including E-cadherin, PTEN and p16^INK4a^ (reviewed in [67]). Interestingly, CtBP appears to play a key role in EBV-mediated transformation through interactions with EBNA 3A and 3C [46], [50]. Mutation of the CtBP binding motifs in EBNA 3A and 3C removes their ability to function as co-operative oncogenes in transformation assays and delays outgrowth of immortalized cells on primary infection [46], [50], [53].The epigenetic repression of p16^INK4a^ by EBNA 3A and 3C via CtBP has recently been shown to be a crucial step in the EBV transformation process [53], [62]. Although our data reveal that *CTBP2* expression is repressed by the EBNA 3 proteins, there are no binding sites for EBNA 2 or 3 proteins at the *CTBP1* locus indicating that *CTBP2* is uniquely targeted. The control of total levels of CtBP levels by modulating *CtBP2* expression may therefore be important in controlling EBV transformation, but it is also possible that CtBP2 could be performing functions distinct from CtBP1 in the cell that may limit immortalization. Recent reports have in fact identified a unique role for CtBP2 as a transcriptional activator [81].

Although we have shown that EBNA 3A, 3B and 3C bind the *CTBP2* intragenic enhancer, our analysis of mRNA expression in EBNA 3 knock-out cell lines to date has only implicated EBNA 3A and EBNA 3B in the repression of *CTBP2* transcription, due to the lack of *CTBP2* expression in the EBNA 3C knock-out cell-lines available. The role of EBNA 3C in the regulation of *CTBP2* expression therefore remains to be tested. Perhaps surprisingly, analysis of cell-lines conditionally expressing EBNA 2 also failed to detect differential regulation of *CTBP2* mRNA levels despite the binding of EBNA 2 to the *CTBP2* enhancer. The EBNA 2 ChIP-seq signal at *CTBP2* is at least half of the signal at the *WEE1* and *ITGAL* sites so it is possible that the affinity of EBNA 2 binding at the *CTBP2* site is lower, making competition for binding with the EBNA 3 proteins less efficient. In EBV-infected cells the binding of the EBNA 3 proteins may therefore be dominant leading to effective inhibition of promoter-enhancer looping.

### WEE1

The *WEE1* gene encodes the Wee1 kinase, a key negative regulator of the G2/M cell-cycle transition [82]. Wee1 inactivates the mitotic cyclin-dependent kinase CDK1 during interphase through phosphorylation on tyrosine 15. This residue is subsequently dephosphorylated by the CDC25 phosphatase at the end of G2, triggering entry into mitosis. EBV infection results in the deregulation of the cell-cycle, with EBNA 3C playing a key role in overriding G1/S, G2/M and mitotic checkpoints through numerous potential mechanisms that include transcriptional repression and the regulation of protein stability [83]–[84]. Disruption of the G2/M checkpoint may be mediated through effects on the Chk2 kinase and we have recently described upregulation of an activator of CDK1, RGC-32, in EBV-infected cells [69], [85]. Although Wee1 has not previously been implicated in EBV-mediated cell-cycle disruption, a recent microarray study identified *WEE1* as the fourth most regulated gene in the ‘cell proliferation’ gene ontology category in newly EBV-infected hyperproliferating B-cells [86]. *WEE1* was upregulated in these infected proliferating cells and subsequently downregulated in the resulting immortalized LCLs, although levels remained higher than in uninfected cells. This pattern of expression was also observed for a key set of DNA damage response genes [86]. Interestingly, EBNA 3C was shown to be required for the repression of the DNA damage response induced in response to EBV infection, thus facilitating the outgrowth of permanently proliferating cells [86]. Our data demonstrating that EBNA 3C can repress *WEE1* expression, an effect counteracted by EBNA 2, may now also implicate EBNA 3C in suppressing the negative effects of *WEE1* on the G2/M checkpoint during the outgrowth of EBV- immortalized cells.

### ITGAL

*ITGAL* encodes alpha L integrin (CD11a) that together with β2 integrin (CD18) forms the heterodimeric integrin, LFA-1 that mediates homotypic and heterotypic adhesion via binding to Intracellular cell adhesion molecules (ICAM) 1–3 [87]. LFA-1 plays an important role in the recruitment of immune cells to sites of infection and promotes T-cell migration and the activation of intracellular signalling cascades [88]–[89]. LFA-1 is upregulated on infection of primary B-cells by EBV along with ICAM1, LFA-3 and a number of B-cell activation molecules, mimicking the B-cell activation phenotype observed on exposure to antigen or mitogens [90]. Expression of the EBV-encoded constitutively active cell-surface receptor LMP1 alone in EBV negative B-cells is sufficient to induce LFA-1 expression on the surface of these cells, implicating LMP1-directed signalling in the activation of *ITGAL* expression [72], [91].

Our analysis has now revealed that EBNA 2 and a cell-type specific subset of EBNA 3 proteins bind to the *ITGAL* promoter *in vivo* implicating the co-ordinated activities of multiple EBV-encoded factors in the regulation of *ITGAL* expression in EBV-infected cells. EBNA 2 and EBNA 3 proteins appear to bind to this site competitively and EBNA 2 activation is inhibited by the presence of any one of the EBNA3s in reporter assays. Since *in vivo* only EBNA 3B binds the *ITGAL* promoter in LCLs and EBNA 3B and EBNA 3C bind in BLs, our reporter assays indicate that when overexpressed all EBNA 3s have the potential to interfere with EBNA 2 activation of the *ITGAL* promoter, presumably through competitive binding. It is therefore likely that the differential *in vivo* binding of the EBNA3s to the *ITGAL* promoter is regulated by the chromatin context and the binding of cellular transcription factors. Interestingly, previous analysis of B-cell lines stably expressing EBNA 2 or EBNA 3C alone reported no change in *ITGAL* (LFA-1) expression [72], [92]. The effects of EBNA 2 and 3C on the *ITGAL* promoter may therefore be context-specific, perhaps depending on the expression of cellular DNA targeting factors and the co-ordinated actions of multiple EBV-encoded latent proteins in infected cells. Consistent with this possibility and similar to *WEE1*, *ITGAL* expression is upregulated in the early stages of proliferation following EBV infection with expression subsequently reduced in the resulting LCLs [86]. Thus *ITGAL* expression may be regulated by LMP1 and the EBNAs in a temporal fashion during infection.

### Mechanism of EBNA 3-mediated repression

Since the anti-EBNA 3C antibody we used for ChIP-seq analysis independently precipitates EBNA 3A and EBNA 3B, our analysis has mapped binding sites for all 3 EBNA 3 proteins in the human genome. Although these sites would be expected to be enriched for EBNA 3C binding sites since the efficiency of EBNA 3A and 3B precipitation by the antibody is lower (Figure S2), we have successfully confirmed EBNA 3A and EBNA 3B binding sites at multiple loci by Q-PCR. Our data thus provide a useful starting point for the study of gene regulation by all three EBNA 3 proteins.

Interestingly, our studies on shared EBNA 2 and EBNA 3 binding sites support a role for EBNA 3 proteins in the repression of all three EBNA target genes we investigated (*CTBP2, WEE1* and *ITGAL*). EBNA 2 appears to compete for binding to these sites limiting EBNA 3-mediated repression to different extents depending on the gene locus. Thus at *CTBP2*, the EBNA 3 proteins appear to inhibit gene activation via the prevention of looping even in the presence of EBNA 2, with loss of EBNA 2 activity having little effect on *CTBP2* gene expression. At *WEE1* however, despite the establishment of chromatin loops that appear to mediate transcriptional repression by EBNA 3C in the presence of EBNA 2, loss of EBNA 2 results in decreased *WEE1* expression indicating that EBNA 2 does limit EBNA 3C-mediated repression to some extent.

Our analysis also demonstrates that repression of gene transcription via binding of EBNA 3 proteins to enhancer elements results from the modulation of chromatin architecture in two different ways. At the *CTBP2* locus, EBNA 3 proteins prevent the formation of chromatin loops that presumably activate the *CTBP2* promoter and at *WEE1*, EBNA 3C is required for the formation of loops associated with transcriptional repression. At an EBNA 3-only binding site at the *ADAM28/ADAMDEC1* locus EBNA 3C again promoted transcriptional repression via enhancer-promoter looping. Interestingly, consistent with our observations at *CTBP2*, the *Drosophila* Snail repressor protein has recently been shown to inhibit transcription via the prevention of enhancer looping (anti-looping) [93]. However, we are unaware of any previous reports of the establishment of chromatin loops by transcriptional repressor proteins that facilitate the repression of target genes. Our results therefore provide important new insights into the mechanism of action of transcriptional repressors.

Our previous analysis correlated binding of EBNA 3C to the intergenic distal binding site between the *ADAM28* and *ADAMDEC1* genes with increases in the polycomb repressor complex-associated H3K27me3 mark across the genes and EBNA 3C-mediated repression of mRNA expression [37]. Consistent with the repressive effects of EBNA 3A on *ADAM28* and *ADAMDEC1* expression [32], [38], our results now indicate that EBNA 3A co-operates in *ADAM* gene repression via association with this intergenic binding site. Thus repression directed via enhancer looping from EBNA 3A and EBNA 3C binding sites to gene promoters appears to be mediated in an analogous fashion to the H3K27me3-associated repression of genes that are targeted via promoter binding sites (*BCL2L11* and p16^INK4a^) [53], [55]. It will be interesting to determine whether EBNA 3C-directed looping at the shared EBNA 2 and 3C *WEE1* enhancer binding sites also directs transcriptional repression via increases in H3K27me3, or through other potential effects on the recruitment or phosphorylation of RNA polymerase II.

### Specificity of EBNA 3A, 3B and 3C targeting and gene regulation

Our studies have uncovered the molecular basis for the regulation of overlapping and distinct sets of cellular genes by different members of the EBNA 3 protein family. This is mediated by gene-specific, and in some cases cell-type specific, differential binding of EBNA 3A, 3B and 3C to gene regulatory elements. This differential binding is evident at both promoter and enhancer binding sites, although we have yet to identify the mechanism through which this differential binding is achieved.

Since EBNA 3 proteins do not bind DNA directly, binding specificity is likely to be directed through interactions with specific B-cell DNA-binding proteins. Unfortunately, analysis of ENCODE ChIP-seq binding data for a vast array of transcription factors was not useful in identifying the specific players involved in targeting EBNA 2 or 3 proteins to DNA. This analysis revealed that a plethora of B-cell transcription factors associate with EBNA 2 and 3 binding sites in LCLs consistent with an extensive network of protein-protein interactions between transcription factors at open chromatin sites. However, using motif searching we found that EBNA 3 binding sites were enriched for PU.1 (Spi-1) binding motifs highlighting an important role for PU.1 in cellular gene targeting by EBNA 3 proteins *in vivo*. PU.1 has been previously implicated in EBNA 3C-mediated co-activation of the LMP1 promoter with EBNA 2 via Spi-1/Spi-B sites [45] and indeed we also found that EBNA 2 and 3 shared sites were enriched for PU.1/Spi-1 binding motifs. Since our ChIP-seq analysis identified EBNA 3A, 3B and 3C binding sites it will be important to determine whether EBNA 3A and 3B can direct cellular gene regulation via PU.1 or whether enrichment of PU.1 binding motifs was detected in our study as a result of its enrichment at EBNA 3C binding sites only. It is of interest that EBNA 3C has recently been shown to bind and stabilise interferon regulatory factor 4 (IRF4) [94], a PU.1 binding partner. Dimerization between the DNA binding domains of PU.1 and IRF4 plays a key role in regulation via adjacent PU.1 and IRF4 motifs, such as those found in the mouse immunoglobulin λ light chain gene enhancer [95]. Although the PU.1-dependent co-activation of the LMP1 promoter by EBNA 3C was not dependent on an adjacent IRF4 binding site [45], it is possible that IRF4 may play a role in directing EBNA 3C-mediated cellular gene regulation, potentially in co-operation with PU.1. It is clear however, that the identification of the factors that direct the specificity of binding of EBNA 3A, 3B and 3C will necessitate a multi-pronged approach using a combination of proteomics to identify chromatin-associated binding partners and analysis of the genome-wide binding profiles of the individual EBNA 3 proteins and any co-associated transcription factor binding.

Although our data indicate that EBNA 2 is not bound to shared sites at the same time as EBNA 3 proteins, we have not been able to definitively determine whether the sets of EBNA 3 proteins bound to the same binding site are associated with the site simultaneously using the currently available antibodies. Recent studies have reported an interaction between EBNA 3A and 3C that could potentially explain the high degree of co-operativity between these two proteins in the regulation of cellular genes if these two proteins are bound to gene regulatory elements as a complex [48]. However, we have identified regulatory sites that are bound by EBNA 3C and not significantly by EBNA 3A at *WEE1* and *ITGAL*, so these two proteins do not always appear to be present at the same site. Examination of the individual binding profiles of EBNA 3A, 3B and 3C and further re-ChIP analysis using new reagents will be required to determine whether these proteins do indeed bind simultaneously.

Given that the remaining EBV-encoded nuclear proteins, EBNA-1 and EBNA-LP also function as transcriptional regulators and multiple EBNA-1 binding sites in the human genome have been mapped in BL cells [96], it will be important in future studies to build up a composite picture of how the EBNAs target the human genome to direct cellular reprogramming by mapping shared and unique binding sites in the same cell background and addressing the combined impact of these factors on cellular gene expression. This will enable us to fully explore the way in which EBV deregulates cellular gene expression to further our understanding of cellular transformation.

## Methods

### Cell lines

The EBV-negative BL cell line DG75 and the EBV-positive latency III BL cell line Mutu III expressing all EBV latent proteins have been described previously [60], [97]. The EBV negative BL31 Burkitt's lymphoma cell lines infected with wild-type recombinant EBV Bacmids or EBNA 2 or EBNA 3C knock-out and revertant Bacmids have been described previously and were kindly provided by Dr R. White and Prof M. Allday [54], [98]. The control (pz1) and EBNA 3C-expressing (E3C-3) stable transfectants of the EBV-negative B-cell lymphoma cell line BJAB were previously described [72]. The sets of wild-type (wt1, wt2, wt3) and EBNA 3A knock-out LCLs (mtB1, mtB2, mtB3) have been previously described and were generated from B-cells derived from two different donors (donor 2 (D2) and donor 3 (D3)) using recombinant viruses [32]. The sets of wild-type and EBNA 3B knock-out LCLs were kindly provided by Dr H. Long and were generated using B-cells from two different donors (PER253 and PER142) infected with either wild-type B95.8 EBV derived from the marmoset B95.8 cell line or EBNA 3B-knock-out recombinant virus [99]. The LCL expressing conditionally active ER-EBNA 2 (ER/EB 2.5) has also been described previously [3]. For β-estradiol withdrawal and add back experiments ER/EB 2.5 cells were incubated in the absence of β-estradiol for 4 days and 1 µM β-estradiol was re-added for 6 or 17 hrs prior to cell harvest. All cell lines were routinely passaged twice-weekly and cultured using the conditions previously described for each line.

### Chromatin immunoprecipitation

For ChIP-sequencing, EBNA 2 and EBNA 3 proteins were immunoprecipitated from 30×10^6^ cross-linked Mutu III cells as described previously [37]. EBNA 3 proteins were precipitated using sheep polyclonal anti-EBNA 3C antibodies (Abcam, ab16128) that we found also independently precipitate EBNA 3A and 3B as a result of cross-reactivity (Figure S2). EBNA 2 was precipitated using a 6-fold scale-up of previously described methods using 48 µg EBNA 2-specific mouse monoclonal antibody (PE2, gift from Prof M. Rowe), followed by an additional incubation with 81 µg rabbit anti-mouse antibodies (Dako) [100]–[101].

For ChIP-Quantitative PCR (ChIP-QPCR) EBNA 2 was immunoprecipiated from 5×10^6^ cross-linked cells using the PE2 monoclonal antibody as described previously [100]–[101]. EBNA 3A ChIP was carried out using 8 µg sheep polyclonal antibodies (Ex-alpha Biologicals, Inc., F115P) and EBNA 3B ChIP was performed using 8 µg sheep polyclonal antibodies (Ex-alpha Biologicals, Inc., F120P) following the protocol previously described for polyclonal antibodies [101]. EBNA 3C ChIP was carried out using 8 µg of E3cD8 monoclonal antibody [102] (gift from Prof M. Rowe) followed by an additional incubation with 13.5 µg rabbit anti-mouse antibodies (Dako).

Re-ChIP was performed essentially as previously described [101] using the antibody quantities described above and BSA-blocked protein A agarose beads (Merck).

### Library preparation, sequencing and data analysis

EBNA 2 ChIP and input DNA was used to generate sequencing libraries that were then subjected to 35 bp single-end read sequencing with an Illumina Genome Analyzer IIx as described previously [37]. ChIP-seq data analysis was carried out as previously described [37] with initial significant peaks of binding identified with MACS (p<10^−7^) [103]. The distance between binding sites and RefSeq gene TSS were calculated and the nearest gene TSS identified. 200 bp regions centred on peak positions were considered as binding sites for further analysis. The distance between binding sites and RefSeq gene TSS were calculated and the nearest gene TSS identified. In unique versus shared site comparisons, for a site to be considered bound by one factor only, the p-value for binding had to be below 10-7 for that factor and above 10-2 for the other factor. For a site to be considered bound by both factors, the p-value for binding had to be below 10-6 for each and the two 200 bp binding regions also had to overlap by at least 100 bp.

### Quantitative real-time PCR

For transcript analysis, total RNA was prepared using Trireagent (Sigma) and purified using RNeasy columns (Qiagen). cDNA was then synthesised using the ImProm-II reverse transcription system and random oligonucleotides (Promega). Real time PCR was carried out using an Applied Biosystems StepOnePlus PCR machine and the primers indicated in Table S1. Real time PCR reactions contained 3 µl of DNA, 7.5 µl of GoTaq qPCR mastermix (Promega) and 2.25 pmoles of each primer in a total volume of 15 µl. DNA was amplified by heating samples to 95°C for 10 minutes followed by 40 cycles of 95°C for 15 seconds and 60°C for 1 minute prior to dissociation curve analysis. Serial dilutions of cDNA or input DNA (for ChIP) were used to generate standard curves for each primer set. For ChIP analysis, the no antibody control signal was subtracted from the percentage input signal derived from the standard curve.

### Chromosome conformation capture

Chromosome conformation capture assays were carried out essentially as described previously [104]. Cells were passed through a 70 µm filter to obtain a single cell preparation. 1×10^7^ cells were then fixed in 1% formaldehyde in the presence of 10% FCS for 10 mins at room temperature. The reaction was quenched with 0.125M glycine, and cells were collected by centrifugation at 230 g at 4°C. The pellet was resuspended in 0.5 ml cold lysis buffer (10 mM Tris-HCl, pH 7.7; 10 mM NaCl; 5 mM MgCl_2_; 0.1 mM EGTA) with freshly added complete protease inhibitors (Roche) and lysed on ice for 10 mins. The nuclei were collected by centrifugation at 400 g for 10 min at 4°C, resuspended in 0.5 ml of 1.2X Buffer 4 (New England Biolabs) containing 0.3% SDS and incubated for 1 hr at 37°C while shaking at 900 rpm. Triton X-100 was then added to the nuclei to give a final concentration of 2% and the samples incubated for 1 hr at 37°C with shaking. 400 U *EcoR*I-HF, *Hind*III or *Aci*I (New England Biolabs) were added to the nuclei and the samples incubated at 37°C overnight with shaking. The digestion reaction was stopped by addition of sodium dodecyl sulphate to a final concentration of 1.6% and incubation at 65°C for 25 mins with shaking. The sample was then diluted 10-fold with 1.15X ligation Buffer (New England Biolabs) containing 1% Triton X-100 and then incubated for 1 hr at 37°C with shaking. 100 U T4 DNA ligase (New England Biolabs) were added to the sample and the reaction was incubated at 16°C for 4 hrs and then 30 mins at room temperature. 300 µg of Proteinase K (Roche) were added to the sample and the reaction incubated at 65°C overnight. RNA was removed by incubation with 300 µg of RNAse for 45 mins at 37°C. Following two rounds of phenol-chloroform extraction, DNA was ethanol precipitated and analysed by PCR using primers designed to amplify across ligation junctions (Table S1).

As a control for ligation products, genomic DNA regions covering restriction sites of interest were amplified by PCR, purified, mixed in equimolar quantities and then digested with *Eco*RI-HF, *Hind*III or *Aci*I (New England Biolabs) for 1.5 hours. Following heat inactivation at 65°C, the digested PCR products were incubated with 10 U T4 DNA-ligase (New England Biolabs) over a temperature range of 4 to 20°C overnight. The DNA was purified using the QIAquick Gel Extraction Kit (Qiagen). Purified positive control DNA was then analysed by PCR using the same primers used for chromosome conformation capture.

### Plasmid construction, transient transfections and reporter assays

The *ITGAL* promoter region from −1756 to +174 bp encompassing all 3 binding sites was amplified from genomic DNA extracted from an LCL using primers designed to introduce *Sac*I and *Hind*III sites at the 5′ and 3′ end respectively (Table S1) and cloned into pGL3 basic (Promega). The EBV C promoter luciferase reporter construct pCp1425GL2 was described previously [105].

DG75 cells were transfected by electroporation as described previously [100]. Cells were transfected with 2 µg pGL3 basic or pGL3-ITGALp, 1 µg of pRL-TK as a transfection control and 10 or 20 µg of pSG5-2A (expressing EBNA 2), pcDNA3 EBNA 3A, EBNA 3B or EBNA 3C expressing constructs (gift from Dr A.Bell). DNA amounts in each transfection were equalized using empty vector. Cells were harvested after 48 hrs and luciferase assays performed using the Dual luciferase assay kit (Promega) as described previously [100] using a Promega Glowmax multidection system.

### Immunoblotting

Immunoblotting was carried out as described previously [100], [105] using the following antibodies: anti-EBNA 3C (mouse monoclonal, E3CA10 [102]), anti-EBNA 3A (sheep polyclonal, Ex-alpha Biologicals, Inc., F115P), anti-EBNA 3B (sheep polyclonal, Ex-alpha Biologicals, Inc., F120P or rabbit polyclonal, Bioss, bs-4698R), anti-EBNA-LP (Santa Cruz biotechnology, Inc sc-23537), anti-actin (Sigma), anti-TFIID (TBP) (Santa Cruz biotechnology, Inc sc-421). EBNA-1 was detected using M.S. human serum as previously described [101].

## Supporting Information

Figure S1 **Western blot analysis of EBNA expression in Mutu III cells and the GM12878 LCL.** EBNA 1, 2, 3A, 3B, 3C and -LP expression was detected by western blotting using whole cell lysates. Blots were probed for actin as a control for loading.
(PDF)

Figure S2 **An EBNA 3C polyclonal antibody independently precipitates EBNA 3A and 3B.** EBNA 3 proteins were immunoprecipitated from stable BJAB transfectants expressing either EBNA 3A (3A-1), EBNA 3B (E3B-2) or EBNA 3C (E3C-3) under the same conditions used for ChIP but in the absence of cross-linking treatment. BJAB cell lysates and immunoprecipitations carried out using EBNA 3A (Ex-alpha F115P), 3B (Ex-alpha F120P) or EBNA 3C (Abcam ab16128) specific antibodies were analysed by Western blotting using EBNA 3A, EBNA 3B or EBNA 3C-specific antibodies. The EBNA 3C antibody is able to independently immunoprecipitate EBNA 3A from cells only expressing EBNA 3A (top panel) and immunoprecipitate EBNA 3B from cells only expressing EBNA 3B (centre panel) indicating that it cross-reacts with these proteins. This EBNA 3C antibody does not however generally cross-react with transcription factors as TATA box binding protein (TBP) is not precipitated.
(PDF)

Figure S3 **Mean histone modification signals at EBNA 2 binding sites.** Aggregate plots of the mean EBNA 2 and EBNA 3 ChIP-seq signals at the top 1000 EBNA 2 binding sites in Mutu III cells compared to ENCODE histone modification ChIP-seq signals in the GM12878 LCL. Each window displays the ChIP-seq signal −/+1 kb around the EBNA 2 binding site midpoint. Dips in the histone modification signal at the binding site midpoint indicate the expected nucleosome-depleted region.
(PDF)

Figure S4 **Mean histone modification signals at EBNA 3 binding sites.** Aggregate plots of the mean EBNA 3 and EBNA 2 ChIP-seq signals at the top 1000 EBNA 3 binding sites in Mutu III cells compared to EBNA 2 and RBP-Jκ ChIP-seq signals in the IB4 LCL [17] and ENCODE transcription factor ChIP-seq signals in the GM12878 LCL (as in Fig. S3).
(PDF)

Figure S5 **EBNA 2 and 3 binding sites are bound by multiple transcription factors.** (A) Heatmap of EBNA 2, EBNA 3 and transcription factor ChIP-seq signals at the top 1000 EBNA 2 binding sites. EBNA 2 and 3 ChIP-seq data from Mutu III BL cells was aggregated with published IB4 EBNA 2 and RBP-Jκ ChIP-seq data and ENCODE GM12878 ChIP-seq data for transcription factors using hierarchical clustering. (B) Heatmap of EBNA 3, EBNA 3and transcription factor ChIP-seq signals at the top 1000 EBNA 3 binding sites. Only transcription factors where significant colocalization with EBNA 2 or 3 sites was observed are shown.
(PDF)

Figure S6 **Mean transcription factor binding signals at EBNA 2 binding sites.** Aggregate plots of the mean EBNA 2 and EBNA 3 ChIP-seq signals at the top 1000 EBNA 2 binding sites in Mutu III cells compared to EBNA 2 and RBP-Jκ ChIP-seq signals in the IB4 LCL [17] and ENCODE transcription factor ChIP-seq signals in the GM12878 LCL (as in Fig. S3).
(PDF)

Figure S7 **Mean transcription factor binding signals at EBNA 3 binding sites.** Aggregate plots of the mean EBNA 3 and EBNA 2 ChIP-seq signals at the top 1000 EBNA 3 binding sites in Mutu III cells compared to EBNA 2 and RBP-Jκ ChIP-seq signals in the IB4 LCL [17] and ENCODE transcription factor ChIP-seq signals in the GM12878 LCL (as in Fig. S3).
(PDF)

Figure S8 **Immunoprecipitation using EBNA 3 knock-out cell lines confirms EBNA 3A, 3B and 3C antibody specificity.** EBNA 3 proteins were immunoprecipitated from BL31 cells infected with wild-type, EBNA 3A KO, EBNA 3B KO or EBNA 3C KO viruses under the same conditions used for ChIP but in the absence of cross-linking treatment. BL31 cell lysates (A, D and G) and immunoprecipitations carried out using EBNA 3A (Ex-alpha F115P), 3B (Ex-alpha F120P) or 3C (E3CD8) specific antibodies were analysed by Western blotting using EBNA 3A (A–C), EBNA 3B (D–F) or EBNA 3C (G–I)-specific antibodies. The EBNA 3A-specific antibody precipitates EBNA 3A from cells infected with wild-type EBV and not EBNA 3A Knock-out EBV (see panel B lanes 2 and 4) (* indicate the position of non-specific bands present in IPs even from knock-out cells). The EBNA 3A antibody does not precipitate EBNA 3B (see panel E lane 4) or EBNA 3C (panel H lane 4) from EBNA 3A Knock-out cells demonstrating that is does not cross-react. The EBNA 3B-specific antibody precipitates EBNA 3B from cells infected with wild-type EBV and not EBNA 3B Knock-out EBV (see panel B lanes 2 and 6) (* indicate the position of non-specific bands present in IPs even from knock-out cells). The EBNA 3B antibody does not precipitate EBNA 3A (panel B lane 6) or EBNA 3C (panel H lane 6) from EBNA 3B Knock-out cells demonstrating that is does not cross-react. The EBNA 3C-specific antibody precipitates EBNA 3C from cells infected with wild-type EBV and not EBNA 3C Knock-out EBV (see panel I lanes 2 and 4). The EBNA 3C antibody does not precipitate EBNA 3A (panel C lane 4) or EBNA 3B (panel F lane 4) from EBNA 3B Knock-out cells demonstrating that is does not cross-react.
(PDF)

Figure S9 **Western blot analysis of EBNA 3A and EBNA 3B knock-out LCLs.** (A) Western blot analysis of EBNA 3A expression in whole cell lysates from wild-type LCLs (wt1, 2 and 3) and LCLs established from EBNA 3A knock-out viruses (mtB1, B2 and B3) in two different donor backgrounds (D2 and D3). The blot was probed for actin as a control for loading. Mutu I and Mutu III cell lysates serve as negative and positive controls, respectively. (B) Western blot analysis of EBNA 3B expression in whole cell lysates from wild-type LCLs infected with B95.8 virus (wt) and EBNA 3B knock-out LCLs (KO) in the PER142 donor background.
(PDF)

Figure S10 ***CTBP2*** **promoter analysis.** Transcript-specific QPCR primers spanning the 6 alternative *CTBP2* transcription start sites displayed in the human genome browser were used to amplify cDNA from wild-type LCLs (D3wt1) where *CTBP2* expression is low and LCLs infected with EBNA 3A Knock-out LCLs (D3mtB1) where *CTBP2* expression is high. Transcripts are named in order of increasing size as (S1, S2, L1, L2, L3 and L4).
(PDF)

Figure S11 **Reciprocal re-ChIP analysis confirms that EBNA 2 and EBNA 3 proteins do not bind simultaneously.** Re-ChIP analysis in Mutu III cells using anti-EBNA 3 antibodies in the first round of ChIP followed by a second round of ChIP in the absence of antibody or using anti-EBNA 2 antibodies. Results show mean percentage primary input −/+ range of two independent Q-PCR reactions from a representative experiment. (A) Re-ChIP analysis at the *CTBP2* enhancer using anti-EBNA 3A antibodies in the first round followed by re-precipitation in the absence of antibody, using anti-EBNA 3A antibodies or anti-EBNA 2 antibodies. (B) Re-ChIP analysis at the *CTBP2* enhancer using anti-EBNA 3B antibodies in the first round followed by re-precipitation in the absence of antibody, using anti-EBNA 3C antibodies or anti-EBNA 2 antibodies. (C) Re-ChIP analysis at the *CTBP2* enhancer using anti-EBNA 3C antibodies in the first round followed by re-precipitation in the absence of antibody, using anti-EBNA 3C antibodies or anti-EBNA 2 antibodies. (D) Re-ChIP analysis at *ITGAL* promoter peak 3 using anti-EBNA 3B antibodies in the first round followed by re-precipitation in the absence of antibody, using anti-EBNA 3B antibodies or anti-EBNA 2 antibodies. (E) Re-ChIP analysis at *ITGAL* promoter peak 3 using anti-EBNA 3C antibodies in the first round followed by re-precipitation in the absence of antibody, using anti-EBNA 3C antibodies or anti-EBNA 2 antibodies. (F) Re-ChIP analysis at *WEE1* enhancer 2 using anti-EBNA 3C antibodies in the first round followed by re-precipitation in the absence of antibody, using anti-EBNA 3C antibodies or anti-EBNA 2 antibodies.
(PDF)

Table S1 **Primers used for chromosome conformation capture, cloning and Q-PCR analysis.**
(PDF)
