# Supplementary figures and images for: Modulation of Enhancer Looping and Differential Gene Targeting by Epstein-Barr Virus Transcription Factors Directs Cellular Reprogramming
Source: PLoS Pathog. 2013 Sep 12;9(9):e1003636. doi: 10.1371/journal.ppat.1003636 (PMC3771879; doi:10.1371/journal.ppat.1003636)

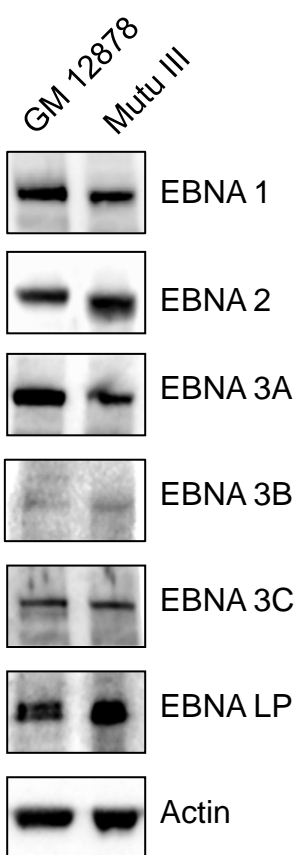

Supplement: Figure S1 — Western blot analysis of EBNA expression in Mutu III cells and the GM12878 LCL. EBNA 1, 2, 3A, 3B, 3C and -LP expression was detected by western blotting using whole cell lysates. Blots were probed for actin as a control for loading. (PDF) [file ppat.1003636.s001.pdf]

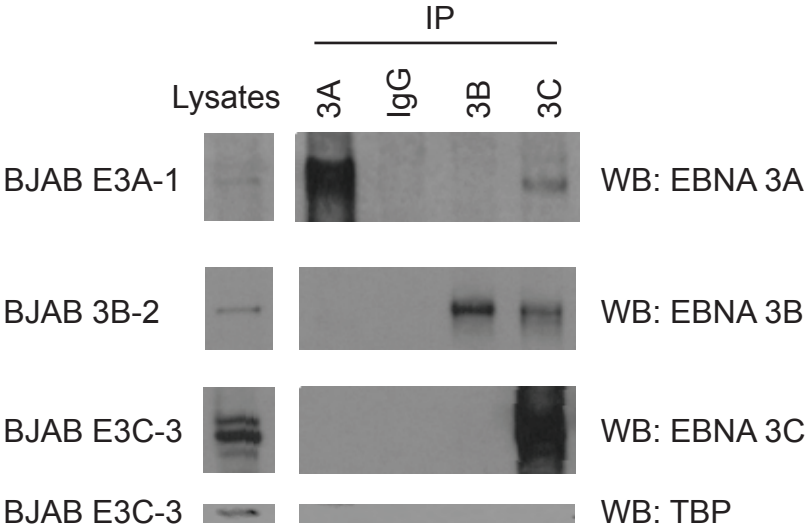

Supplement: Figure S2 — An EBNA 3C polyclonal antibody independently precipitates EBNA 3A and 3B. EBNA 3 proteins were immunoprecipitated from stable BJAB transfectants expressing either EBNA 3A (3A-1), EBNA 3B (E3B-2) or EBNA 3C (E3C-3) under the same conditions used for ChIP but in the absence of cross-linking treatment. BJAB cell lysates and immunoprecipitations carried out using EBNA 3A (Ex-alpha F115P), 3B (Ex-alpha F120P) or EBNA 3C (Abcam ab16128) specific antibodies were analysed by Western blotting using EBNA 3A, EBNA 3B or EBNA 3C-specific antibodies. The EBNA 3C antibody is able to independently immunoprecipitate EBNA 3A from cells only expressing EBNA 3A (top panel) and immunoprecipitate EBNA 3B from cells only expressing EBNA 3B (centre panel) indicating that it cross-reacts with these proteins. This EBNA 3C antibody does not however generally cross-react with transcription factors as TATA box binding protein (TBP) is not precipitated. (PDF) [file ppat.1003636.s002.pdf]

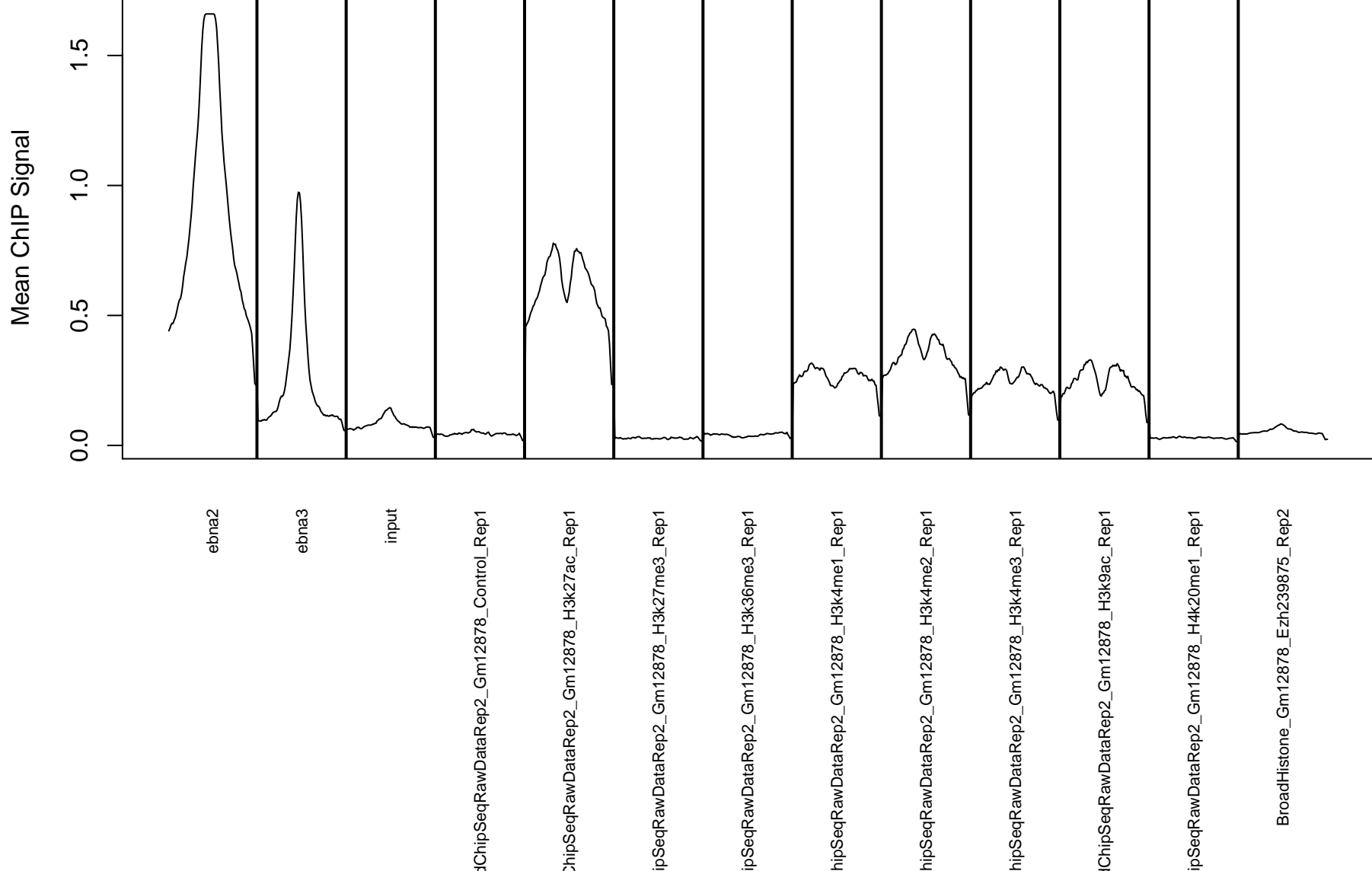

Supplement: Figure S3 — Mean histone modification signals at EBNA 2 binding sites. Aggregate plots of the mean EBNA 2 and EBNA 3 ChIP-seq signals at the top 1000 EBNA 2 binding sites in Mutu III cells compared to ENCODE histone modification ChIP-seq signals in the GM12878 LCL. Each window displays the ChIP-seq signal −/+1 kb around the EBNA 2 binding site midpoint. Dips in the histone modification signal at the binding site midpoint indicate the expected nucleosome-depleted region. (PDF) [file ppat.1003636.s003.pdf]

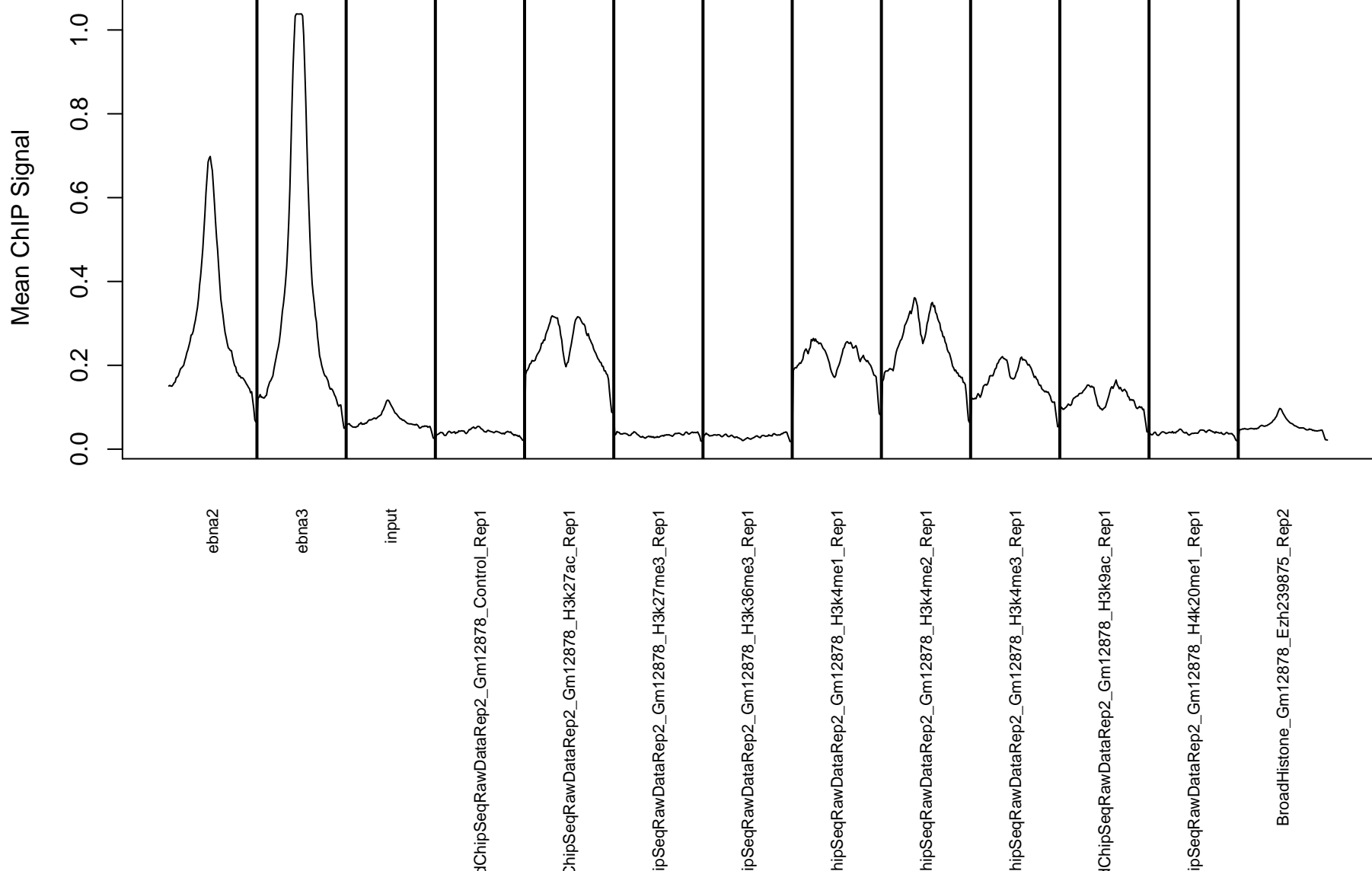

Supplement: Figure S4 — Mean histone modification signals at EBNA 3 binding sites. Aggregate plots of the mean EBNA 3 and EBNA 2 ChIP-seq signals at the top 1000 EBNA 3 binding sites in Mutu III cells compared to EBNA 2 and RBP-Jκ ChIP-seq signals in the IB4 LCL [17] and ENCODE transcription factor ChIP-seq signals in the GM12878 LCL (as in Fig. S3). (PDF) [file ppat.1003636.s004.pdf]

**A**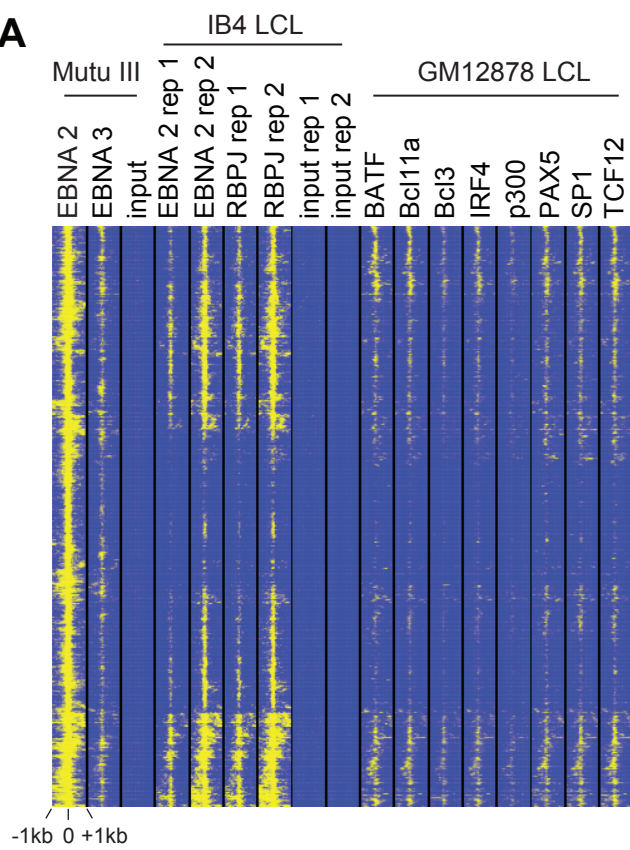**B**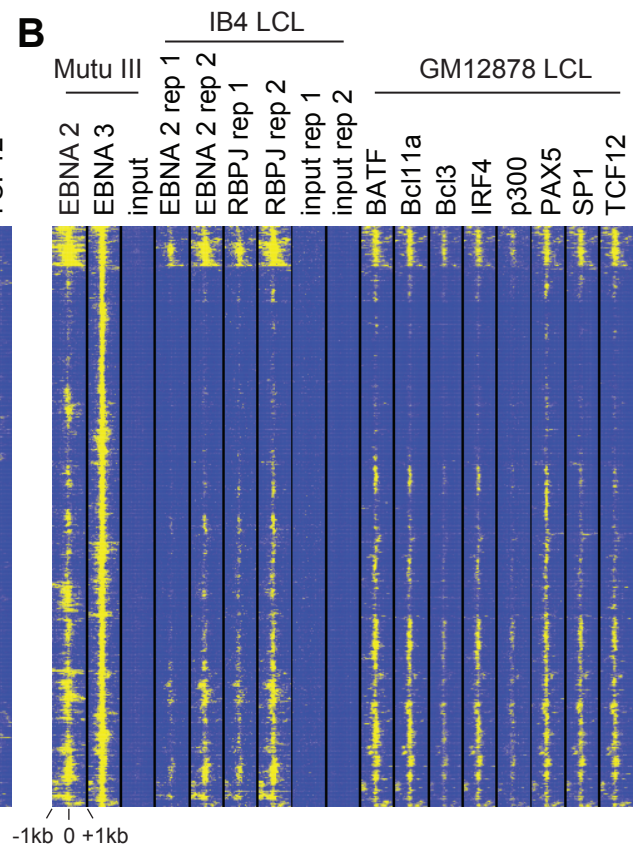

Supplement: Figure S5 — EBNA 2 and 3 binding sites are bound by multiple transcription factors. (A) Heatmap of EBNA 2, EBNA 3 and transcription factor ChIP-seq signals at the top 1000 EBNA 2 binding sites. EBNA 2 and 3 ChIP-seq data from Mutu III BL cells was aggregated with published IB4 EBNA 2 and RBP-Jκ ChIP-seq data and ENCODE GM12878 ChIP-seq data for transcription factors using hierarchical clustering. (B) Heatmap of EBNA 3, EBNA 3and transcription factor ChIP-seq signals at the top 1000 EBNA 3 binding sites. Only transcription factors where significant colocalization with EBNA 2 or 3 sites was observed are shown. (PDF) [file ppat.1003636.s005.pdf]

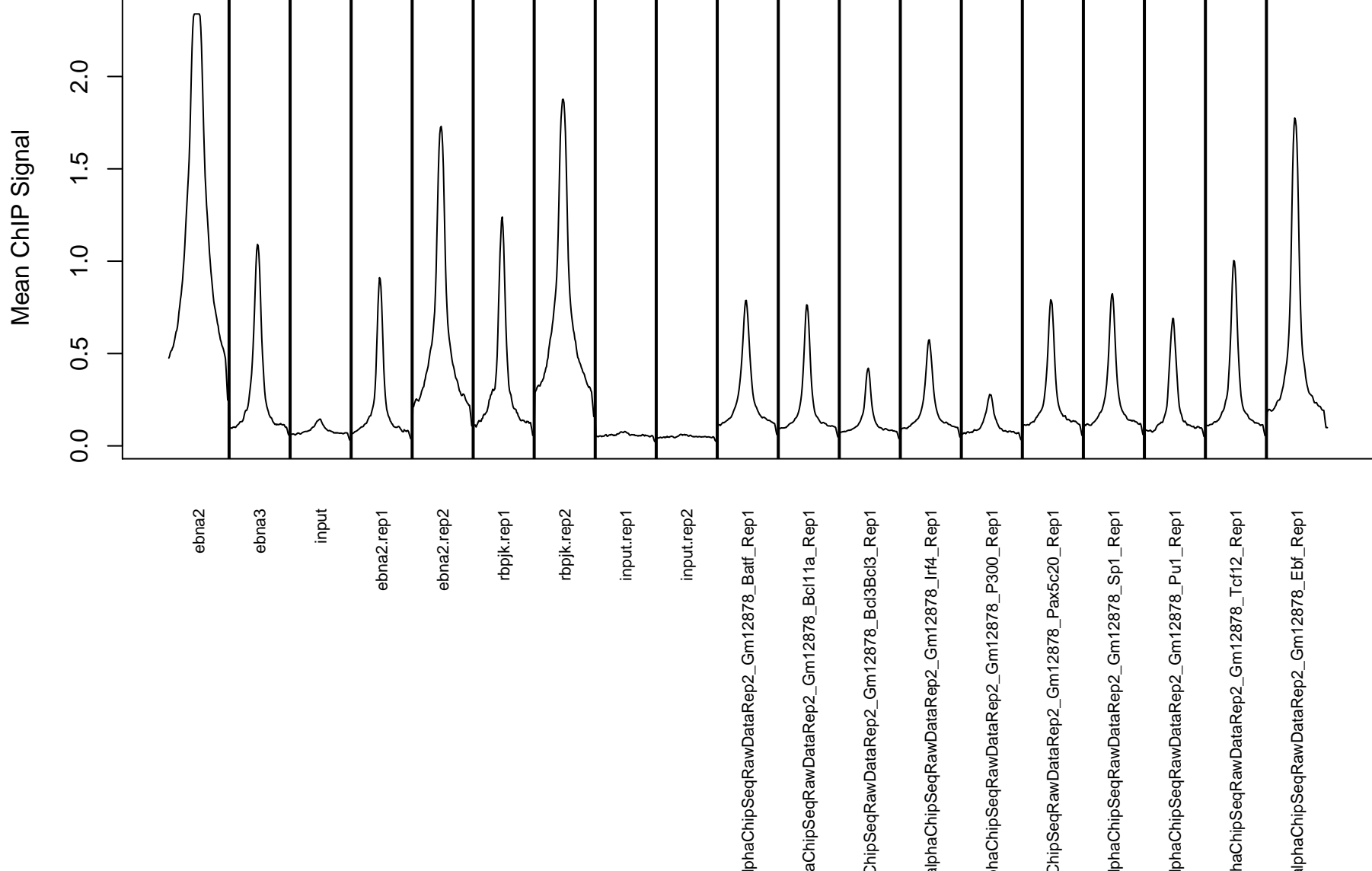

Supplement: Figure S6 — Mean transcription factor binding signals at EBNA 2 binding sites. Aggregate plots of the mean EBNA 2 and EBNA 3 ChIP-seq signals at the top 1000 EBNA 2 binding sites in Mutu III cells compared to EBNA 2 and RBP-Jκ ChIP-seq signals in the IB4 LCL [17] and ENCODE transcription factor ChIP-seq signals in the GM12878 LCL (as in Fig. S3). (PDF) [file ppat.1003636.s006.pdf]

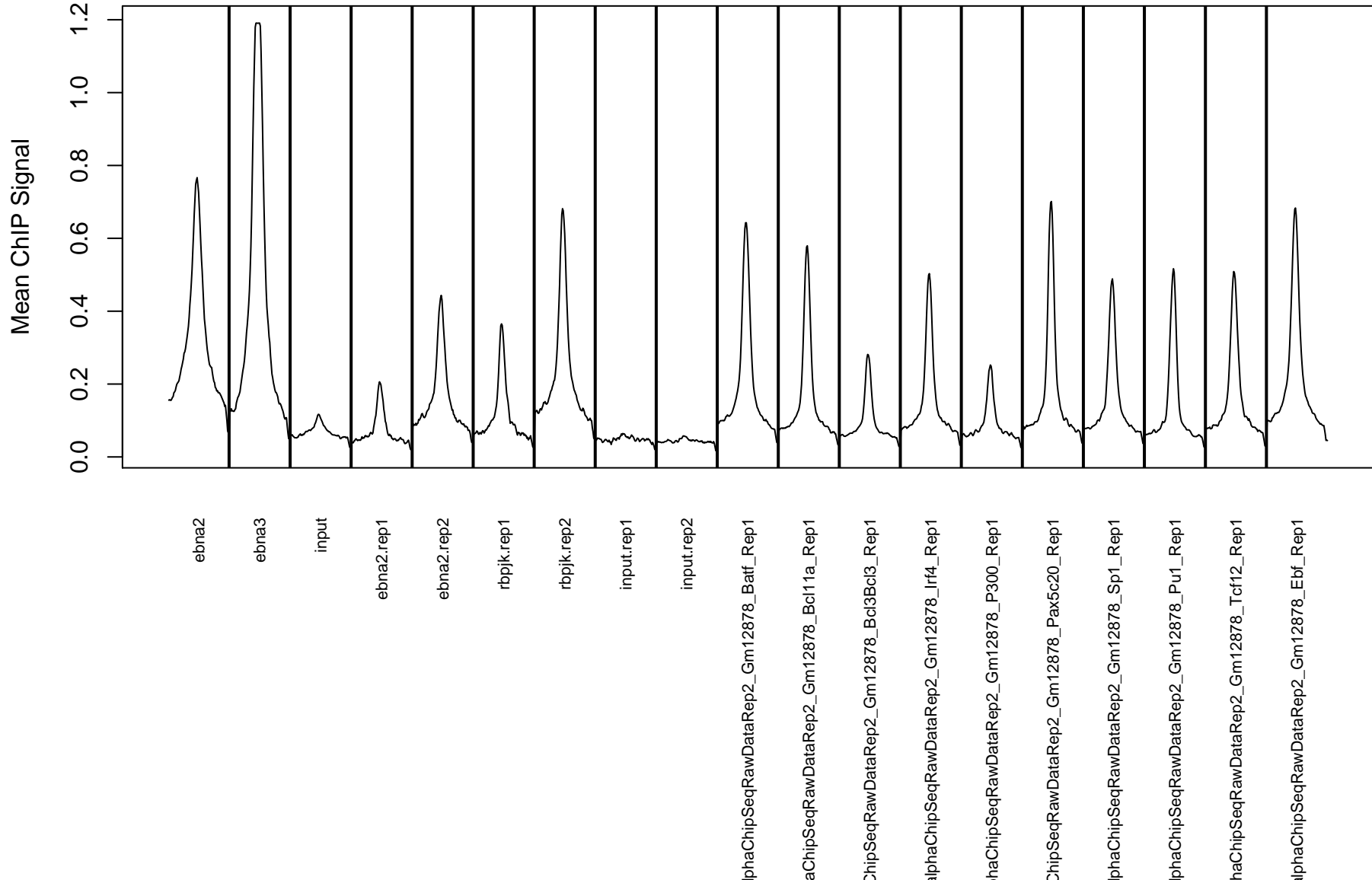

Supplement: Figure S7 — Mean transcription factor binding signals at EBNA 3 binding sites. Aggregate plots of the mean EBNA 3 and EBNA 2 ChIP-seq signals at the top 1000 EBNA 3 binding sites in Mutu III cells compared to EBNA 2 and RBP-Jκ ChIP-seq signals in the IB4 LCL [17] and ENCODE transcription factor ChIP-seq signals in the GM12878 LCL (as in Fig. S3). (PDF) [file ppat.1003636.s007.pdf]

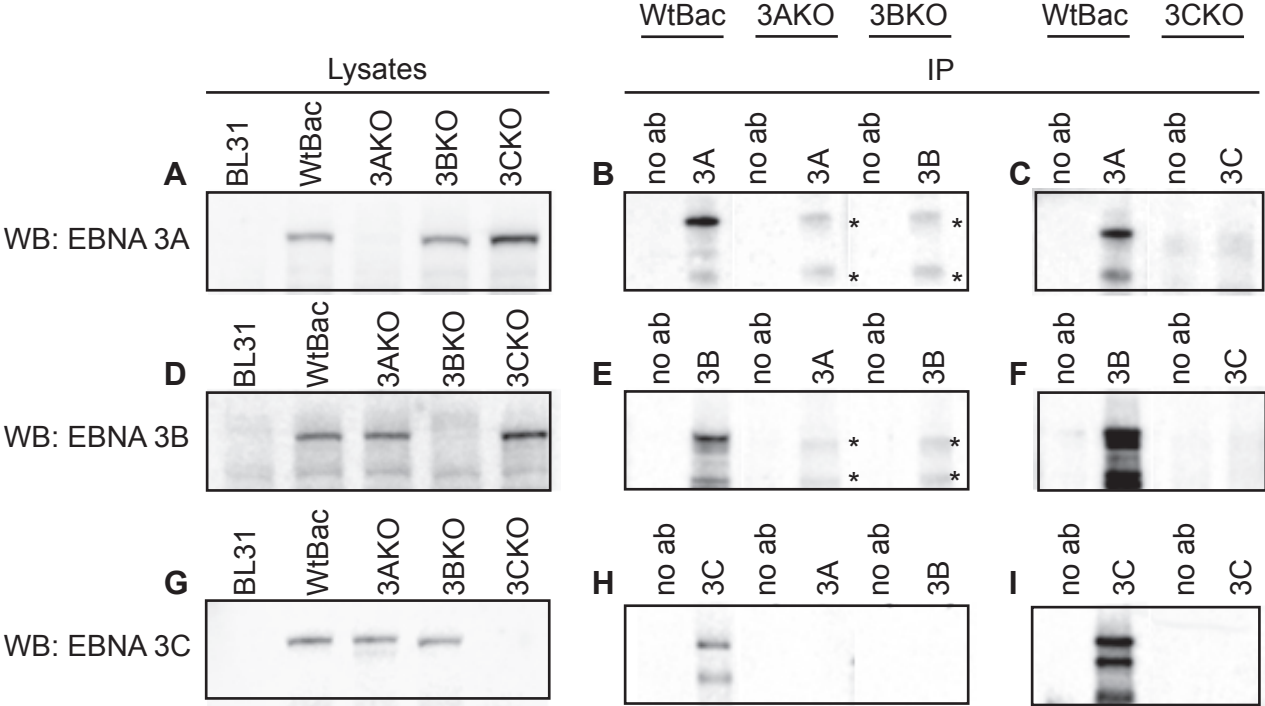

Supplement: Figure S8 — Immunoprecipitation using EBNA 3 knock-out cell lines confirms EBNA 3A, 3B and 3C antibody specificity. EBNA 3 proteins were immunoprecipitated from BL31 cells infected with wild-type, EBNA 3A KO, EBNA 3B KO or EBNA 3C KO viruses under the same conditions used for ChIP but in the absence of cross-linking treatment. BL31 cell lysates (A, D and G) and immunoprecipitations carried out using EBNA 3A (Ex-alpha F115P), 3B (Ex-alpha F120P) or 3C (E3CD8) specific antibodies were analysed by Western blotting using EBNA 3A (A–C), EBNA 3B (D–F) or EBNA 3C (G–I)-specific antibodies. The EBNA 3A-specific antibody precipitates EBNA 3A from cells infected with wild-type EBV and not EBNA 3A Knock-out EBV (see panel B lanes 2 and 4) (* indicate the position of non-specific bands present in IPs even from knock-out cells). The EBNA 3A antibody does not precipitate EBNA 3B (see panel E lane 4) or EBNA 3C (panel H lane 4) from EBNA 3A Knock-out cells demonstrating that is does not cross-react. The EBNA 3B-specific antibody precipitates EBNA 3B from cells infected with wild-type EBV and not EBNA 3B Knock-out EBV (see panel B lanes 2 and 6) (* indicate the position of non-specific bands present in IPs even from knock-out cells). The EBNA 3B antibody does not precipitate EBNA 3A (panel B lane 6) or EBNA 3C (panel H lane 6) from EBNA 3B Knock-out cells demonstrating that is does not cross-react. The EBNA 3C-specific antibody precipitates EBNA 3C from cells infected with wild-type EBV and not EBNA 3C Knock-out EBV (see panel I lanes 2 and 4). The EBNA 3C antibody does not precipitate EBNA 3A (panel C lane 4) or EBNA 3B (panel F lane 4) from EBNA 3B Knock-out cells demonstrating that is does not cross-react. (PDF) [file ppat.1003636.s008.pdf]

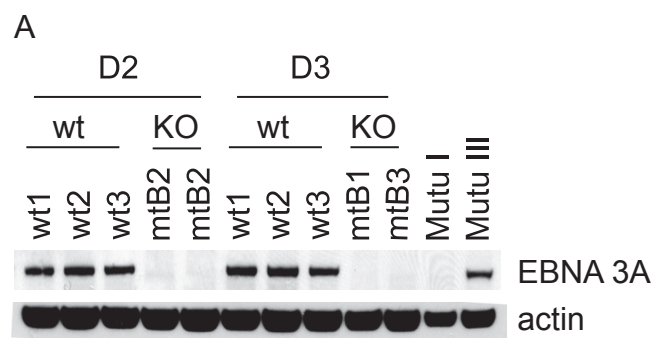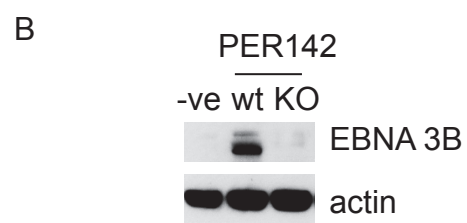

Supplement: Figure S9 — Western blot analysis of EBNA 3A and EBNA 3B knock-out LCLs. (A) Western blot analysis of EBNA 3A expression in whole cell lysates from wild-type LCLs (wt1, 2 and 3) and LCLs established from EBNA 3A knock-out viruses (mtB1, B2 and B3) in two different donor backgrounds (D2 and D3). The blot was probed for actin as a control for loading. Mutu I and Mutu III cell lysates serve as negative and positive controls, respectively. (B) Western blot analysis of EBNA 3B expression in whole cell lysates from wild-type LCLs infected with B95.8 virus (wt) and EBNA 3B knock-out LCLs (KO) in the PER142 donor background. (PDF) [file ppat.1003636.s009.pdf]

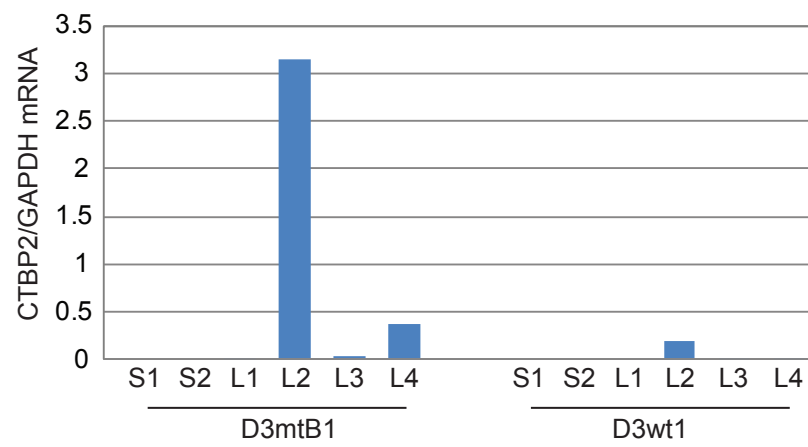

Supplement: Figure S10 — CTBP2 promoter analysis. Transcript-specific QPCR primers spanning the 6 alternative CTBP2 transcription start sites displayed in the human genome browser were used to amplify cDNA from wild-type LCLs (D3wt1) where CTBP2 expression is low and LCLs infected with EBNA 3A Knock-out LCLs (D3mtB1) where CTBP2 expression is high. Transcripts are named in order of increasing size as (S1, S2, L1, L2, L3 and L4). (PDF) [file ppat.1003636.s010.pdf]

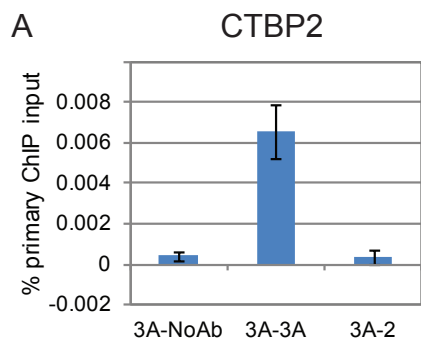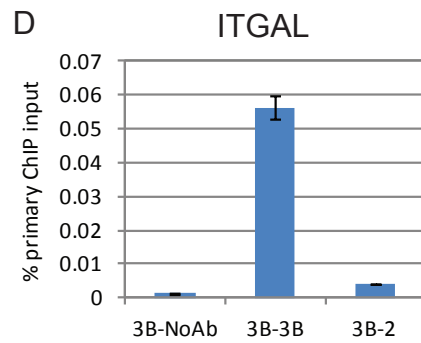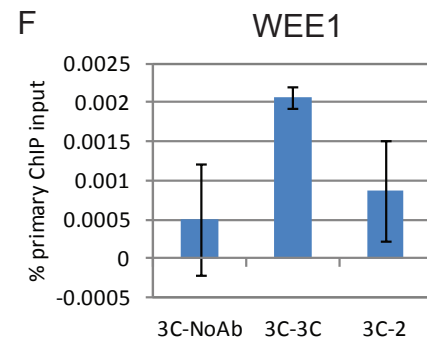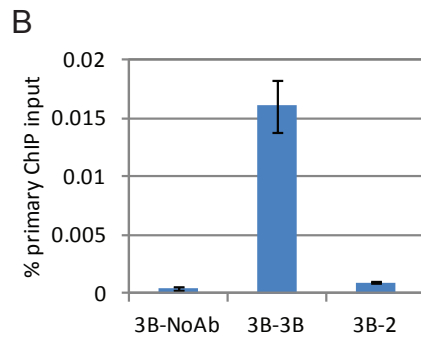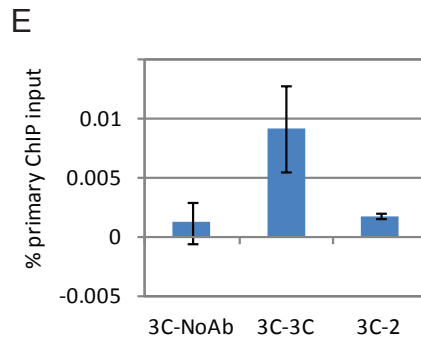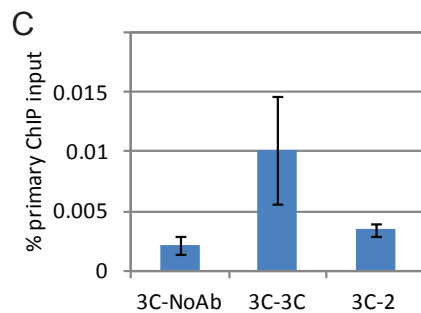

Supplement: Figure S11 — Reciprocal re-ChIP analysis confirms that EBNA 2 and EBNA 3 proteins do not bind simultaneously. Re-ChIP analysis in Mutu III cells using anti-EBNA 3 antibodies in the first round of ChIP followed by a second round of ChIP in the absence of antibody or using anti-EBNA 2 antibodies. Results show mean percentage primary input −/+ range of two independent Q-PCR reactions from a representative experiment. (A) Re-ChIP analysis at the CTBP2 enhancer using anti-EBNA 3A antibodies in the first round followed by re-precipitation in the absence of antibody, using anti-EBNA 3A antibodies or anti-EBNA 2 antibodies. (B) Re-ChIP analysis at the CTBP2 enhancer using anti-EBNA 3B antibodies in the first round followed by re-precipitation in the absence of antibody, using anti-EBNA 3C antibodies or anti-EBNA 2 antibodies. (C) Re-ChIP analysis at the CTBP2 enhancer using anti-EBNA 3C antibodies in the first round followed by re-precipitation in the absence of antibody, using anti-EBNA 3C antibodies or anti-EBNA 2 antibodies. (D) Re-ChIP analysis at ITGAL promoter peak 3 using anti-EBNA 3B antibodies in the first round followed by re-precipitation in the absence of antibody, using anti-EBNA 3B antibodies or anti-EBNA 2 antibodies. (E) Re-ChIP analysis at ITGAL promoter peak 3 using anti-EBNA 3C antibodies in the first round followed by re-precipitation in the absence of antibody, using anti-EBNA 3C antibodies or anti-EBNA 2 antibodies. (F) Re-ChIP analysis at WEE1 enhancer 2 using anti-EBNA 3C antibodies in the first round followed by re-precipitation in the absence of antibody, using anti-EBNA 3C antibodies or anti-EBNA 2 antibodies. (PDF) [file ppat.1003636.s011.pdf]
