# Supplementary material for: Modulation of Enhancer Looping and Differential Gene Targeting by Epstein-Barr Virus Transcription Factors Directs Cellular Reprogramming
Source: PLoS Pathog. 2013 Sep 12;9(9):e1003636. doi: 10.1371/journal.ppat.1003636 (PMC3771879; doi:10.1371/journal.ppat.1003636)
Supplement: Table S1 — Primers used for chromosome conformation capture, cloning and Q-PCR analysis. (PDF) [file ppat.1003636.s012.pdf]

Primer Table	
**primer name**	**primer sequence 5' to 3'**
**ChIP primers**	
**WEE1**	
WEE1 A fw MW798	CAAGCCATCTTCACACCTCA
WEE1 A rv MW799	TTGAGACCAGCCTAGACAACAA
WEE1 B fw MW800	TGCTAAGTGGATGAGGCAGA
WEE1 B rv MW801	TGCACAGCTGCATTCCTC
WEE1 C fw MW802	TTGCTGCAACACCAGCTC
WEE1 C rv MW803	CACCAGGAACACTGGAGACA
WEE1 D fw MW804	TCCAGACAGCACCAGGAAG
WEE1 D rv MW805	CTGTGGGAAAGTCCTCCAGT
WEE1 E fw MW806	TACAGGCGTGAGCCACTG
WEE1 E rv MW807	GTCTGCACCAAGTGACAAGC
WEE1 F fw MW808	GGGACTCCTGGTGTTAGTCCT
WEE1 F rv MW809	CATAGAAGGAGGCCTGTCAAA
WEE1 G fw MW810	ACCACTGCAGGAAGCGTTAT
WEE1 G rv MW811	GATTCGTGCACATCTGTTGAA
WEE1 H fw MW812	ACCCGGTCCTAACTGGAGA
WEE1 H rv MW813	GAGCCTCGCTCCAGAGACT
WEE1 I fw MW814	GGACATTGTATTTATTCAGGTCCA
WEE1 I rv MW815	GCTCCCTGAATCCTATCCACT
WEE1 J fw MW816	CCCTGGGTTAGTCATGCAA
WEE1 J rv MW817	GACTCCCATGTGTGGTTGG
**ITGAL**	
ITGAL A fw MW820	CAGGCTGGAGTACAGTGGTG
ITGAL A rv MW821	GAGGCTGAGGCAGGAGAAT
ITGAL B fw MW822	TGTAAAGGGTATCTCACTGTGGTT
ITGAL B rv MW823	ATAAATGGCCAACATGCACA
ITGAL C fw MW824	AGGCTGGTCTCGAACTCTTG
ITGAL C rv MW825	CGGTGGCTCACACCTCTAA

ITGAL D fw MW826	TGCACCTGTGGTTTCAGCTA
ITGAL D rv MW827	CGATCACAGCTCAATGCAAC
ITGAL E fw MW828	ACCCAGCCTCCAATTCTTTAG
ITGAL E rv MW829	TTTCTCTGGACCTTGAAAGATGT
ITGAL F fw MW830	TGCTTACACTTCCTCCCTGAA
ITGAL F rv MW831	TTTCTCACAGAGGCAACAGG
**CTBP2**	
CTBP2 A fw MW834	TGGCTATGTCACAGCGATTC
CTBP2 A rv MW835	ACCGTGCCTGATGGAGTT
CTBP2 B fw MW836	TTGCATATTTGGGATTTCAGTTT
CTBP2 B rv MW837	TTCTGTGTGAAACAGTTGTGGTT
CTBP2 C fw MW838	CCAGGACAACGTCTGAGTGA
CTBP2 C rv MW839	CCATCGCCATGTTAACAGAA
**BCL2L11**	
BCL2L11 A fw MW870	CAGAGGGAGGAGAGCTCAAA
BCL2L11 A rv MW871	GAGTTTCTAAGCCGCTCTGG
BCL2L11 B fw MW876	CTGGTGAAGGGTCGTAGGTC
BCL2L11 B rv MW877	CCCGATACTACGAGCAGGTC
BCL2L11 C fw MW878	AGGACAAGTGGCGAGGACT
BCL2L11 C rv MW879	GCCAGTCACCTGGAGACAA
**Cloning primers**	
**ITGAL**	
ITGAL peaks Ampl. fw MW848	GTATGAGCTCGAGAATGACTCGAGCCCGTGAG
ITGAL peaks Ampl. rv MW849	GCATAAGCTTTTCCAGCACTCGAGGGACC
**cDNA primers**	
**WEE1**	
WEE1 cDNA ex8-9 fw MW818	TGAAGAGGGCGATAGTCGTT
WEE1 cDNA ex8-9 rv MW819	CACCAGCAGCACATACCACT

**ITGAL**	
ITGAL cDNA ex25-26 fw MW832	CCAAGTCAAGCACATGTACCA
ITGAL cDNA ex25-26 rv MW833	ACGCTCCACTGGTGTGTG
**CTBP2**	
CTBP2 cDNA ex5-6 fw MW840	GAGTGATCGTGCGGATAGG
CTBP2 cDNA ex5-6 rv MW841	GAGTCCGCTGTCTCTTCCAC
**CCC primers**	
**WEE1**	
WEE1 P to E1 fw MW 888	GTGAAGAGGCTGGATGGATG
WEE1 P to E1 rv MW 889	GCTGGAGTACAGTGGCACAA
WEE1 P to E2 fw MW 890	AATCGGCTCTGGAGAATTTG
WEE1 P to E2 rv MW 891	CCTGGGTGAGAGCAAGACTC
WEE1 P to Con fw MW 892	TGCCATTAAGCGATCAAAAA
WEE1 P to Con rv MW 893	TCAGGCCCTCCTTTCTTGTA
**CTBP2**	
CTBP2 P to E1 fw MW 952	CCTGGGGGTTTCTTATCCAT
CTBP2 P to E1 rv MW 953	GGACCCCACGTTTAGGATTT
CTBP2 P to Con fw MW 954	GGCCGTTATCCACCTCTTCT
CTBP2 P to Con rv MW 953	GGACCCCACGTTTAGGATTT
**ADAM locus**	
ADAM 28 P to E1 fw MW901	CCAGTTTGGCTCTTTCTGTTTGAG
ADAM 28 P to E1 rv MW902	GCTCCCATGCCACATAAAACTTG
ADAM 28 P to Con 1 fw MW901	CCAGTTTGGCTCTTTCTGTTTGAG
ADAM 28 P to Con 1 rv MW903	ACTTCTGTAGGTGGATGTTACTGG
ADAM 28 P to Con 2 fw MW901	CCAGTTTGGCTCTTTCTGTTTGAG
ADAM 28 P to Con 2 rv MW904	GGACTAGCGATACAACTTTACGG
ADAMDEC1 P to E1 fw MW913	ACCTAACATTTTCCCTCCTTCC
ADAMDEC1 P to E1 rv MW915	TCCAATCATCCACCTTTCTTCC
ADAMDEC1 P to Con fw MW913	ACCTAACATTTTCCCTCCTTCC
ADAMDEC1 P to Con rv MW959	CCATTGTGGAATACCATGTGG
